# Evaluation of a serum-based antigen test for tuberculosis in HIV-exposed infants: a diagnostic accuracy study

**DOI:** 10.1186/s12916-021-01983-w

**Published:** 2021-05-18

**Authors:** Liyan Mao, Sylvia M. LaCourse, Soyeon Kim, Chang Liu, Bo Ning, Duran Bao, Jia Fan, Christopher J. Lyon, Ziyong Sun, Sharon Nachman, Charles D. Mitchell, Tony Y. Hu

**Affiliations:** 1 Center for Cellular and Molecular Diagnostics, Biochemistry and Molecular Biology, Tulane University School of Medicine, Room 474, 333 S. Liberty Street, New Orleans, LA 70112 USA; 2 Department of Laboratory Medicine, Tongji Hospital, Tongji Medical College, Huazhong University of Science and Technology, Wuhan, 430030 China; 3 Departments of Medicine and Global Health, Division of Allergy and Infectious Diseases, University of Washington, Seattle, WA 98104 USA; 4 Frontier Science Foundation, Brookline, MA 02115 USA; 5 Department of Chemical Engineering, Biomedical Engineering Program, University of South Carolina, Columbia, SC 29208 USA; 6 Department of Pediatrics, State University of New York at Stony Brook, Stony Brook, NY 11794 USA; 7 Department of Pediatrics, Division of Infectious Diseases and Immunology, University of Miami Miller School of Medicine, Batchelor Children’s Research Institute, Room 286, 1580 NW 10th Avenue, Miami, FL 33136 USA

**Keywords:** Pediatric tuberculosis, CFP-10, Nanotechnology, Mass spectrometry

## Abstract

**Background:**

Non-sputum methods are urgently needed to improve tuberculosis diagnosis and treatment monitoring in children. This study evaluated the ability of a serum assay quantifying a species-specific peptide of the *Mycobacterium tuberculosis* CFP-10 virulence factor via nanotechnology and matrix-assisted laser desorption ionization time-of-flight mass spectrometry to diagnose tuberculosis in HIV-infected and HIV-uninfected infants.

**Methods:**

Serum CFP-10 peptide signal was blinded evaluated in cryopreserved sera of 519 BCG-immunized, HIV-exposed infants (284 HIV-infected, 235 HIV-uninfected) from a multi-center randomized placebo-controlled isoniazid prophylaxis trial conducted in southern Africa between 2004 and 2008, who were followed up to 192 weeks for *Mtb* infection and TB. Children were classified as confirmed, unconfirmed, or unlikely tuberculosis cases using 2015 NIH diagnostic criteria for pediatric TB.

**Results:**

In HIV-infected infants, CFP-10 signal had 100% sensitivity for confirmed TB (5/5, 95% CI, 47.8–100) and 83.7% sensitivity for unconfirmed TB (36/43, 95% CI 69.3–93.2), with 93.1% specificity (203/218, 95% CI 88.9–96.1). In HIV-uninfected infants, CFP-10 signal detected the single confirmed TB case and 75.0% of unconfirmed TB cases (15/20; 95% CI 50.9–91.3), with 96.2% specificity (177/184, 95% CI, 92.3–98.5). Serum CFP-10 achieved 77% diagnostic sensitivity for confirmed and unconfirmed TB (13/17, 95% CI, 50–93%) at ≤ 24 weeks pre-diagnosis, and both CFP-10-positivity and concentration declined following anti-TB therapy initiation.

**Conclusions:**

Serum CFP-10 signal exhibited high diagnostic sensitivity and specificity for tuberculosis in HIV-infected and HIV-uninfected infants and potential utility for early TB detection and monitoring of anti-TB treatment responses.

**Supplementary Information:**

The online version contains supplementary material available at 10.1186/s12916-021-01983-w.

## Background

Approximately one million children develop tuberculosis (TB) and 205,000 die of TB-related causes each year [1]. Eighty percent of these deaths occur in children < 5 years old, with the majority (96%) of deaths occurring among children who did not receive treatment [2], where missed diagnoses are likely responsible for undertreatment. Children with TB, particularly infants, frequently have paucibacillary TB, exhibit non-specific symptoms, and are likely to rapidly progress to disseminated or extrapulmonary TB in the absence of appropriate treatment [3–6]. This clinical presentation, combined with difficulty obtaining respiratory samples, makes it challenging to diagnose pediatric TB and monitor treatment responses using standard sputum-based methods [3, 7, 8].

The gold-standard of mycobacterial culture is positive in only 30–62% of pediatric TB cases [8, 9]. Molecular diagnostic assays such as Xpert MTB/RIF and Xpert MTB/RIF Ultra enable rapid diagnosis but are less sensitive in children [10–14]. Immunological tests (e.g., tuberculin skin tests (TSTs) and interferon gamma-release assays) detect the immune response to *Mycobacterium tuberculosis (Mtb)*, but do not distinguish *Mtb* infection from TB, and are less sensitive in children with compromised or immature immune systems [15, 16]. However, direct detection of circulating *Mtb*-derived factors, such as the virulence factors CFP-10 and ESAT-6 [17], can be hampered by low levels and masking effects [18], and current direct detection assays have demonstrated poor sensitivity in identifying active TB [19–21].

We recently developed a novel assay applying nanotechnology and matrix-assisted laser desorption ionization time-of-flight (MALDI-TOF) mass spectrometry to directly detect an *Mtb-*specific CFP-10 peptide (CFP-10pep) in trypsin-digested sera, allowing TB diagnosis from a small blood volume (100 μL). This assay diagnosed pulmonary TB with high sensitivity in culture-confirmed HIV-negative (93%) and HIV-positive (91%) adults (specificity 87–100%) and detected serum CFP-10pep in extrapulmonary and culture-negative TB cases [18, 22, 23]. This assay uses fresh or frozen serum, allowing samples to be transported to and analyzed at central testing sites without restricting the time available to obtain valid assay results. Non-sputum diagnostic methods that utilize small blood volumes could be of particular benefit to infants, in whom sputum confirmation is typically lacking at diagnosis and during treatment. Herein, we evaluated the diagnostic performance of this assay for pediatric TB, using stored sera from HIV-exposed infants enrolled in a TB prevention trial.

## Methods

### Participants and study design

De-identified serum and clinical data were obtained from IMPAACT P1041, a multi-center randomized, double-blind, placebo-controlled trial conducted in southern Africa between 2004 and 2008 that compared pre-exposure isoniazid (INH) prevention therapy to placebo in infants with perinatal HIV exposure [24]. Infants meeting P1041 enrollment criteria (see Additional file) were randomized to daily INH or placebo for 96 weeks after obtaining informed consent from their legal guardians, segregated into HIV-infected and HIV-uninfected subgroups, and evaluated for up to 96 weeks after intervention. HIV-infected infants were scheduled for study visits every 12 weeks, but HIV-uninfected infant visits were reduced to every 24 weeks after their intervention period. Infants were evaluated for TB by the P1041criteria at all visits using all diagnostic evidence (see Additional file), and sera remaining from blood assays was archived.

P1041 participants were eligible for the current study if they had complete demographic information, results from all conducted clinical tests, and at least one analyzable serum sample after excluding hemolyzed, hyperlipidemic, and low volume (< 100 μL) samples. Serum was analyzed by personnel who were blinded to all clinical and TB classification data using a previously reported assay employing nanoparticle-based immune-enrichment and MALDI-TOF mass spectrometry to detect and quantify CFP-10pep signal, as described below. Statistical analyses were performed using data from a locked database containing clinical information and CFP-10pep results. All study personnel committed to the research plan and to ensuring the accuracy and completeness of the data analysis.

### Case classification

Infants were categorized post-hoc by investigators with pediatric TB expertise (CDM, SML) blinded to CFP-10pep results using 2015 NIH consensus criteria for pediatric TB diagnosis (hereafter 2015 NIH criteria) [25]. Infants were categorized as confirmed TB (*Mtb* culture-positive), unconfirmed TB (≥ 2 non-bacteriologic forms of TB evidence), or unlikely TB (no/insufficient evidence of TB or a confirmed alternative diagnosis) [25]. Unconfirmed and unlikely TB cases were categorized by the presence or absence of immunologic evidence of *Mtb* infection (TST result; see Additional file). Extrapulmonary TB cases were identified using the P1041 criteria [ 24].

### Serum CFP-10pep assays

Serum CFP-10pep was analyzed using a nanoparticle-based immunoenrichment assay read by MALDI-TOF mass spectrometry (see Additional file) that detects *Mtb*-specific CFP-10pep from trypsin-digested serum or EDTA plasma samples [18, 22, 23]. Samples producing CFP-10pep peaks (m/z 1593.75) with signal-to-noise ratios ≥ 3 were considered CFP-10pep-positive, and CFP-10pep signal was analyzed as a ratio against a spiked sequence-matched isotope-labeled internal standard (IS) peptide (m/z 1603.60).

CFP-10pep diagnostic sensitivity estimates utilized the serum sample collected nearest TB diagnosis and within ± 24 weeks of diagnosis by 2015 NIH criteria (± 1 HIV-uninfected cohort post-intervention study visit), since few infants had serum available at their post-hoc diagnosis visit. CFP-10pep diagnostic specificity estimates analyzed all available serum from unlikely TB cases, and participants were considered CFP-10pep-negative only if *all* their samples were CFP-10pep-negative.

CFP-10pep changes pre-TB diagnosis and post-treatment initiation were analyzed using all samples from confirmed and unconfirmed TB cases, grouping CFP-10pep results by 12-week intervals, and comparing CFP-10pep changes among TB cases with and without positive treatment responses (resolution/improvement of TB signs/symptoms within 3 months of treatment initiation without new signs/symptoms).

### Statistical analyses

Statistical analyses employed IBM SPSS version 24.0, and GraphPad Prism 7 for chi-square and Fisher exact tests, one-way ANOVAs with Bonferroni’s post-tests, and Kruskal–Wallis one-way ANOVAs with Dunn’s post-tests, as determined by sample distribution and variance. Sensitivity and specificity are reported with Clopper-Pearson 95% CIs. Differences were considered statistically significant for two-sided tests with *p* values < 0.05.

## Results

### Study population and baseline characteristics

**Fig. 1:**
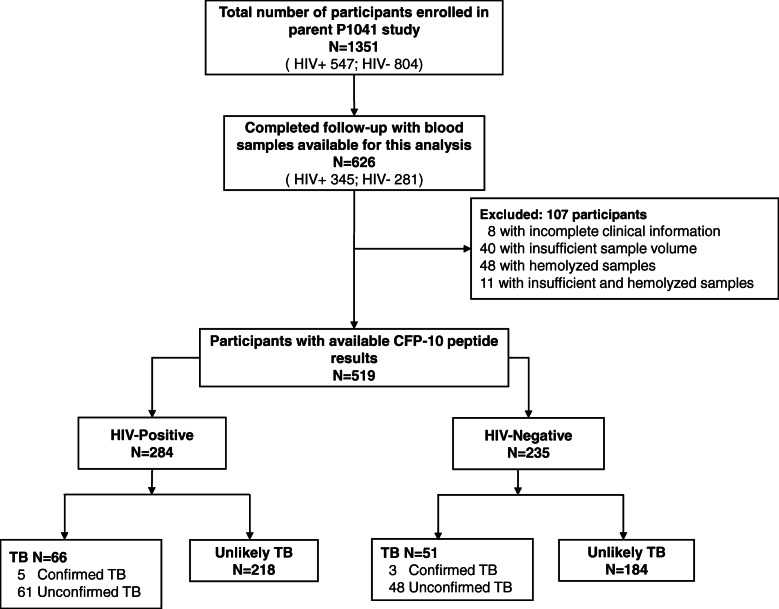
Inclusion of P1041 parent study participants with evaluable clinical information and serum samples. All children that met inclusion criteria were classified using 2015 NIH criteria for TB diagnosis into one of three groups: (1) confirmed TB: all children with a positive *Mtb* culture result; (2) unconfirmed TB: all children with ≥ 2 different categories of non-bacteriological evidence of TB; and (3) unlikely TB (non-TB): all children with no evidence, or insufficient evidence, of TB or a confirmed alternative diagnosis

Of the 1351 enrolled P1041 infants, 626 completed follow-up and had archived serum samples, and 519 remained after excluding all those with missing clinical information or with unanalyzable serum samples (Fig. 1). Differences between included and excluded infants were primarily minor (Additional file: Table S1), although HIV-infected children were excluded at higher rates and included children exhibited higher rates of TB regardless of their HIV infection status. Median age at enrollment was 94 days, 45.3% were HIV-infected, and 46.6% were male (Table 1). Half the infants diagnosed with TB by 2015 NIH criteria developed TB by 15 months of age (median 48 weeks post-enrollment; Additional file: Fig. S1A), which differed between HIV-infected and HIV-uninfected infants (median 36 versus 72 weeks, *p* = 0.008; Additional file: Fig. S1B-C).

**Table 1:** Baseline demographics and clinical characteristics of study participants by HIV and TB status

	HIV-infected^**a**^			HIV-uninfected		
	Total (*N* = 284)	TB^b^ (*N* = 66)	Unlikely TB^c^ (*N* = 218)	Total (*N* = 235)	TB^b^ (*N* = 51)	Unlikely TB^c^ (*N* = 184)
Age—days						
Median	95	96	95	93^#^	93	93^#^
Range	91 to 120	91 to 120	91 to 120	91 to 120	91 to 119	91 to 120
WHO weight-for-age *z* score						
Median	− 1.35	− 1.39	− 1.10	− 0.33^#^	− 0.46^#^	− 0.30^#^
Range	− 5.84 to 3.48	− 5.84 to 2.17	− 5.84 to 3.48	− 4.96 to 1.98	− 4.96 to 1.87	− 3.34 to 1.98
Male sex—no. (%)	116 (40.8)	29 (42.4)	87 (39.9)	126 (53.6)^#^	27 (52.9)^#^	99 (53.8)^#^
Race or ethnic group—no. (%)						
Indigenous African	283 (99.6)	66 (100.0)	217 (99.5)	234 (99.6)	51 (100.0)	183 (99.5)
Mixed ancestry or other	1 (0.4)	0 (0.0)	1 (0.5)	1 (0.4)	0 (0.0)	1 (0.5)
Breast-feeding—no. (%)						
Ever breast-fed	42 (14.8)	6 (9.1)	36 (16.5)	19 (8.1)^#^	2 (4.0)	17 (9.2)^#^
Breast-fed at baseline	22 (7.7)	4 (6.1)	18 (8.3)	3 (1.3)^#^	0 (0.0)	3 (1.6)^#^
Parent study arm—no. (%)^d^						
Isoniazid	150 (52.8)	33 (50.0)	117 (53.7)	113 (48.1)	23 (45.1)	90 (48.9)
Placebo	134 (47.2)	33 (50.0)	101 (46.3)	122 (51.9)	28 (54.9)	94 (51.1)
Maternal history of tuberculosis—no. (%)	13 (4.6)	5 (7.6)	8 (3.7)	15 (6.8)	3 (5.9)	12 (6.5)
During index pregnancy	1 (0.4)	1 (1.5)	0	1(0.4)	0	1 (0.5)
Before index pregnancy	12 (4.2)	4 (6.1)	8 (3.7)	14 (6.4)	3 (5.9)	11 (6.0)
CDC clinical HIV category—no. (%)^e^						
N (asymptomatic)	223 (79.6)	53 (81.5)	170 (79.1)	–	–	–
A (mildly symptomatic)	43 (15.4)	9 (13.9)	34 (15.8)	–	–	–
B (B moderately symptomatic)	14 (5.0)	3 (4.6)	11 (5.1)	–	–	–
CD4+ percentage—%						
Median	30	25	30^ǂ^	–	–	–
Range	6 to 58	9 to 48	6 to 58	–	–	–
CD4+ percentage category—no. (%)^f^						
< 20%	52 (19.7)	14 (23.7)	38 (17.7)	–	–	–
20–24%	33 (12.5)	14 (23.7)	29 (13.5)	–	–	–
25–34%	104 (39.4)	21 (35.6)	83 (38.6)	–	–	–
≥ 35%	75 (28.4)	10 (16.9)	65 (30.2)	–	–	–
Plasma HIV-1 RNA at entry—copies/ml						
Median	527,000	750,000	386,000^ǂ^	–	–	–
Range	≤ 400 to > 750,000	≤ 400 to > 750,000	≤ 400 to > 750,000	–	–	–
TB related parameters—n/N (%)^g^						
MTB culture	5/111 (4.5)	5/61 (8.2)	0/50 (0.0)	5/68 (7.4) ^i^	5/42 (11.9)	0/26 (0.0)
AFB smear	14/114 (12.3)	9/64 (14.1)	5/50 (10.0)	9/70 (12.9)	6/44 (13.6)	3/26 (11.5)
TST	78/202 (38.6)	46/66 (69.7)	32/136 (23.5)^ǂ^	51/226 (22.6)^#^	30/51 (58.8)	21/175 (12.0)^#ǂ^
TB contact exposure	39/41 (95.1)	27/28 (96.4)	12/13 (92.3)	34/38 (89.5)	21/24 (87.5)	13/14 (92.9)
Chest X-ray	75/134 (56.0)	58/66 (87.9)	17/68 (25.0)^ǂ^	61/98 (62.2)	49/51 (96.1)	12/47 (25.5)^ǂ^
Signs/symptoms consistent with TB	13/284 (4.6)	10/66 (15.2)	3/218 (1.4)^ǂ^	11/235 (4.7)	6/51 (11.8)	5/184 (2.7)^ǂ^
Positive response to anti-TB therapy^h^	38/77 (49.4)	37/60 (61.7)	1/17 (5.9)^ǂ^	33/61 (54.1)	32/46 (69.6)	1/15 (6.7)^ǂ^

HIV status was determined following enrollment; TB status was retroactively applied to the study population using the 2015 NIH criteria

Symbols denote *p* values < 0.05 for differences between the TB and unlikely TB groups (ǂ) and the HIV-infected and HIV-uninfected groups (#)

^a^Two participants were HIV-uninfected at entry but tested HIV-infected approximately 24 weeks after enrollment and therefore classified as HIV-infected

^b^Participants with confirmed TB (*Mtb* culture-positive) and unconfirmed TB (≥ 2 types of non-bacteriological evidence of TB) evaluated as a group since the size of the confirmed TB group (*N* = 8; see Fig. 1) prevent meaningful comparisons with other groups

^c^Unlikely TB: children never suspected of TB or suspected of TB but with no, or insufficient, evidence for TB diagnosis, or with a confirmed alternative diagnosis

^d^Children enrolled in the P1041 parent study received INH prophylaxis for up to 96 weeks or until achieving a primary endpoint (first occurrence of TB disease or *Mtb* infection, or death from any cause) to evaluate whether primary INH prophylaxis improved TB disease-free survival among HIV-infected children or *Mtb* infection-free survival among HIV-uninfected children in a population immunized with the BCG vaccine

^e^Four participants with missing data and HIV-uninfected participants at entry were not included from the percentages

^f^20 participants with missing data and HIV-uninfected participants at entry were not included from the percentages

^g^n/N (%) indicate the number of children with positive results per the total number of children with this information and the percent of children with a positive result

^h^Children classified as TB cases (definite, probable, and possible TB) according to the P1041 protocol or as having “non-algorithm TB” by clinical experts were started on anti-TB treatment. The majority of children identified as TB cases in the P1041 study were also categorized as TB cases by the 2015 NIH criteria (Table S10)

^i^Two TB patients with culture positive results were first diagnosed as unconfirmed TB

The proportion of TB cases (confirmed and unconfirmed) was similar between HIV-infected and HIV-uninfected infants (23.2% versus 21.7%, *p* = 0.75) as were diagnosis distributions (Table 2). Most TB cases were pulmonary TB, but five HIV-infected infants had extrapulmonary TB, with two having both pulmonary and extrapulmonary TB. The distribution of infants who received INH or placebo did not differ by HIV status and TB category (Additional file: Table S2). Most TB cases (86.3%, 101/117) had *Mtb* culture, smear, chest X-ray, and TST data, but were primarily diagnosed by chest X-ray and TST (Additional file: Fig. S2).

**Table 2:** Summary of groups present in current study

	Total * (***N*** = 519)	HIV-infected (***N*** = 284)	HIV-uninfected (***N*** = 235)	***P*** value
**TB cases—no. (%)**	117 (22.5)	66 (23.2)*	51 (21.7)	0.68
**Confirmed PTB**	6 (1.2)	3 (1.1)	3 (1.3)	0.26
**Confirmed EPTB** ^**a**^	2 (0.4)	2 (0.7)	–	–
**Unconfirmed PTB**	108 (20.8)	60 (21.1)	48 (20.4)	–
MTB infection ^**b**^	73 (14.1)	45 (15.8)	28 (11.9)	0.10
No MTB infection ^**c**^	35 (6.7)	15 (5.3)	20 (8.5)	–
**Unconfirmed EPTB** ^**d**^	3 (0.6)	3 (1.1)	–	–
**Unlikely TB**—**no. (%)**	402 (77.5)	218 (76.8)	184 (78.3)	–
MTB infection	53 (10.2)	32 (11.3)	21 (8.9)	0.33
No MTB infection ^**e**^	349 (67.3)	186 (65.5)	163 (69.4)	–
**Died**	9 (1.7)	7 (2.5)	2 (0.9)	0.16

*Two infants diagnosed with both unconfirmed EPTB and unconfirmed PTB were counted once in this total

^a^Both of these EPTB cases were diagnosed as TB lymphadenitis

^b^MTB infection = TST-positive (induration of ≥ 5 mm for HIV-infected infants and ≥ 10 mm for HIV-uninfected infants)

^c^No MTB infection = TST-negative (induration of < 5 mm for HIV-infected infants and < 10 mm for HIV-uninfected infants)

^d^Two infants were diagnosed with both unconfirmed TB lymphadenitis and unconfirmed PTB, and the third was diagnosed with TB meningitis

^e^TST-negative or no TST result available for infants not suspected of TB who did not reach a protocol-designated visit for TST performance (parent study weeks 96, 144, 196)

### CFP-10pep diagnostic performance for pediatric TB

Samples suitable for CFP-10pep analysis were limited in number and distribution (Additional file: Table S3 and Table S4), and few infants had serum available at their retrospective diagnosis by 2015 NIH criteria (14% and 8% of HIV-infected and HIV-uninfected infants). We therefore analyzed the serum sample that was dawn nearest TB diagnosis (± 1 post-intervention study visit for the HIV-uninfected cohort) to calculate CFP-10pep diagnostic sensitivity, since most confirmed (75%; 6/8) and unconfirmed (57.8%; 63/109) TB cases had serum within this interval. Children with and without serum in this window revealed similar clinical characteristics (Additional file: Table S5 and Fig. S2), suggesting the absence of selection bias.

Serum CFP-10pep demonstrated 95% overall specificity (22/402, Table 3), which ranged from 85.7 to 100% in the unlikely TB subgroups (Table 4), and robust sensitivity for confirmed (100%; 6/6) and unconfirmed (81%; 51/63) TB cases diagnosed by clinical evidence (Table 3; Additional file: Table S6). HIV-infected and HIV-uninfected infants with unconfirmed TB had similar CFP-10pep diagnostic sensitivities (84% versus 75%; *p* = 0.50) and specificities (93% versus 96%, *p* = 0.19), which differed in HIV-uninfected infants only with evidence of *Mtb* infection (86% versus 98%, *p* = 0.03, Table 3). CFP-10pep was not detected in most unlikely TB cases with evidence of *Mtb* infection (88·7%), including HIV-infected (90·6%) and HIV-uninfected (85·7%) individuals (Table 3). INH prophylaxis did not appear to affect CFP-10pep sensitivity for confirmed and unconfirmed TB or specificity for unlikely TB, when analyzed overall or by HIV- or *Mtb*-infection status (Additional file: Tables S7; Table S8).

**Table 3:** Diagnostic performance of CFP-10pep assay for TB

	Confirmed TB^**α**^	Unconfirmed TB^**β**^			Unlikely TB^**γ**^		
		MTB infection^a^	No MTB infection^b^	Total	MTB infection	No MTB infection^c^	Total
**All participants (*N*** **= 471)**							
CFP-10pep +, no. (%)	6 (100.0)	34 (79.1)	17 (85.0)	51 (81.0)	6 (11.3)	16 (4.6)	22 (5.5)
CFP-10pep −, no. (%)	–	9 (20.9)	3 (15.0)	12 (19.0)	47 (88.7)	333 (95.4)	380 (94.5)
Sensitivity—% (95% CI)	100 (54.1–100.0)	79.1 (64.0–90.0)	85.0 (62.1–96.8)	81.0 (69.1–89.8)	–	–	–
Specificity—% (95% CI)	–	–	–	–	88.7 (77.0–95.7)	95.4 (92.7–97.4)	94.5 (91.8–96.5)
**HIV-infected participants (*N*** **= 266)**							
CFP-10pep +, no. (%)	5 (100.0)	24 (80.0)	12 (92.3)	36 (83.7)	3 (9.4)	12 (6.4)	15 (7.7)
CFP-10pep −, no. (%)	–	6 (20.0)	1 (7.7)	7 (16.3)	29 (90.6)	174 (93.6)	203 (92.3)
Sensitivity—% (95% CI)	100 (47.8–100.0)	80.0 (61.4–92.3)	92.3 (64.0–99.8)	83.7 (69.3–93.2)	–	–	–
Specificity—% (95% CI)	–	–	–	–	90.6 (75.0–98.0)	93.6 (89.0–96.6)	93.1 (88.9–96.1)
**HIV-uninfected participants (*N*** **= 205)**							
CFP-10pep +, no. (%)	1 (100.0)	10 (76.9)	5 (71.4)	15 (75.0)	3 (14.3)	4 (2.4)	7 (4.3)
CFP-10pep −, no. (%)	–	3 (23.1)	2 (28.6)	5 (25.0)	18 (85.7)	159 (97.6)	177 (96.2)
Sensitivity—% (95% CI)	100 (2.5–100.0)	76.9 (46.2–95.0)	71.4 (29.0–96.3)	75.0 (50.9–91.3)	–	–	–
Specificity—% (95% CI)	–	–	–	–	85.7 (63.7–97.0)	97.6 (93.8–99.3)	96.2 (92.3–98.5)

*CI* confidence interval

^α^Confirmed TB: *Mtb* culture-positive

^β^Unconfirmed TB: ≥ 2 types of non-bacteriological TB evidence within 12 weeks

^γ^Unlikely TB: no or insufficient evidence of TB or a confirmed alternative diagnosis (did not have active TB)

^a^TST positive (induration of ≥ 5 mm for HIV-infected children and ≥ 10 mm for HIV-uninfected children)

^b^TST negative (induration of < 5 mm for HIV-infected children and < 10 mm for HIV-uninfected children)

^c^TST-negative or no TST result available for children not suspected of TB who did not reach a protocol-designated visit for TST performance (parent study weeks 96, 144, 196)

CFP-10pep diagnostic sensitivity was calculated using the sample drawn closest (± 24 weeks; ± 1 study visit for the HIV-uninfected cohort following INH/placebo treatment) to the time of their retrospective diagnosis by 2015 NIH criteria

**Table 4:** CFP-10pep specificity in subgroups of the unlikely TB population

Unlikely TB	Total		HIV-infected		HIV-uninfected	
	CFP-10 (pos./total)	Specificity % (95%CI)	CFP-10 (pos./total)	Specificity % (95%CI)	CFP-10 (pos./total)	Specificity % (95%CI)
NTM infection	2/14	85.7 (57.2–98.2)	1/12	91.7 (61.5–99.8)	1/2	50.0 (1.3–98.7)
Other infections*	13/153	91.5 (85.9–95.4)	11/88	87.5 (78.7–93.6)	2/65	96.9 (89.3–99.6)
Other inflammatory diseases	7/148	95.3 (90.5–98.1)	3/63	95.2 (86.7–99.0)	4/85	95.3 (88.4–98.7)
Other diseases	0/35	100 (90.0–100)	0/28	100 (87.7–100)	0/7	100 (59.0–100)
Latent TB infection	5/44	88.6 (75.4–96.2)	3/25	88.0 (68.8–97.5)	2/19	89.5 (66.9–98.7)
No disease (healthy)	1/45	97.8 (88.2–99.9)	1/27	96.3 (81.0–99.9)	0/18	100 (81.5–100)
Unclear diagnosis	0/5	100 (47.8–100)	0/1	100 (2.5–100)	0/4	100 (39.8–100)
Total	22/402^#^	94.5 (91.8–96.5)	15/218	93.1 (88.9–96.1)	7/184	96.2 (92.3–98.5)

*Bacterial, viral, fungal, and other infections

^#^Several children had co-infections, including 7 with non-tuberculous mycobacterium (NTM) infections and 12 with latent TB infections, while 23 children with latent TB infections also had an unrelated inflammatory disease

**Fig. 2:**
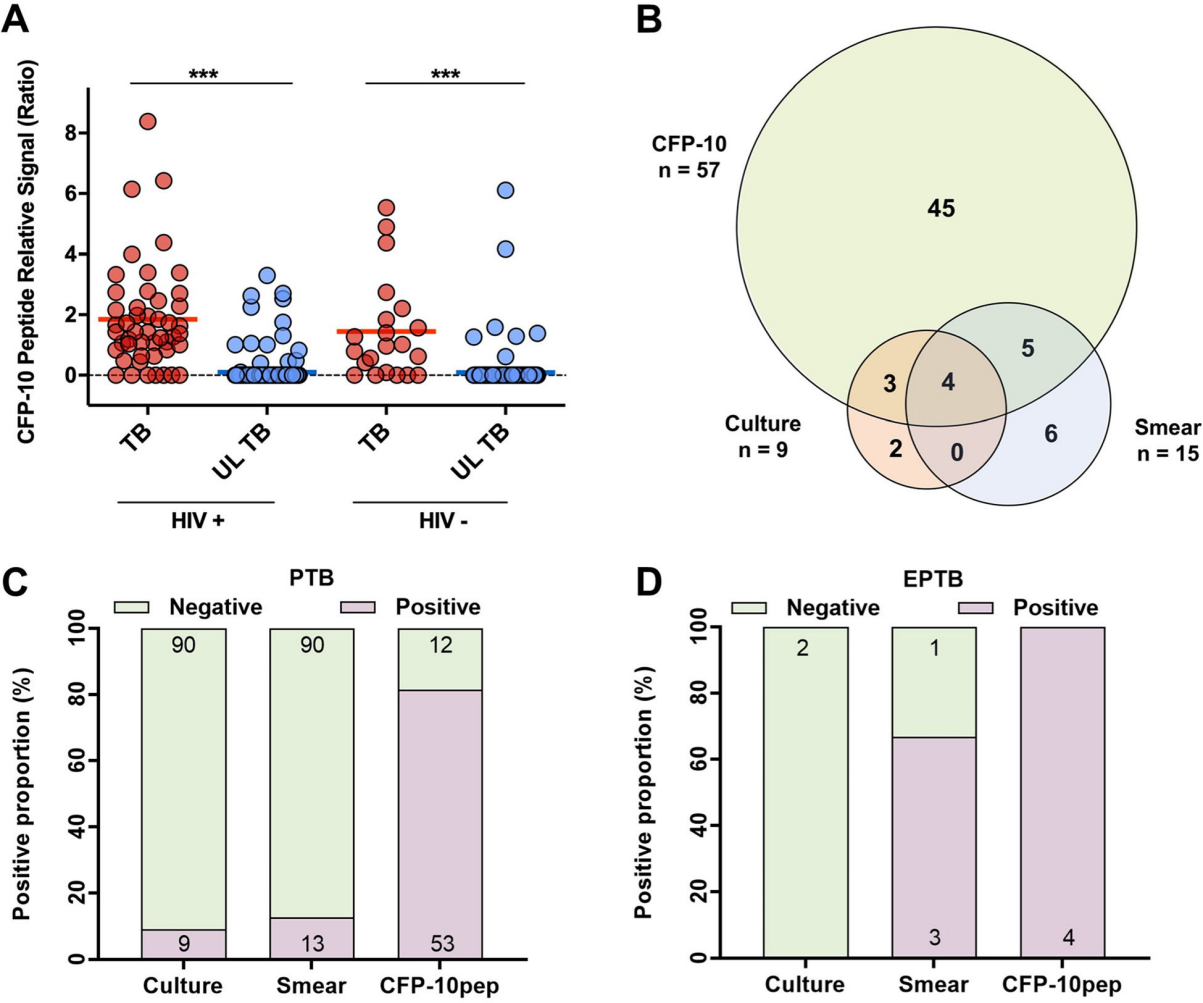
Serum CFP-10 can distinguish TB from unlikely TB cases and the comparison with other methods. **a** Serum CFP-10 signal in HIV-infected and HIV-uninfected children with TB and unlikely (UL) TB, where horizontal bars indicate mean values. ***p < 0.001 by ANOVA with Tukey’s post-test. **b** Venn diagram of all positive CFP-10pep, and *Mtb* culture and smear results. Note that one child diagnosed with unconfirmed TB subsequently had a positive Mtb culture, resulting in a mismatch between the overall number of confirmed TB cases and positive *Mtb* cultures. The positive proportions of culture, smear and CFP-10pep among extrapulmonary TB **c** PTB only and **d** EPTB cases

CFP-10pep signal was higher in TB versus unlikely TB cases in both HIV-infected (1.9 ± 0.3 versus 0.1 ± 0.03, *p* < 0.001) and HIV-uninfected infants (1.4 ± 0.4 versus 0.09 ± 0.04, *p* < 0.001; Fig. 2a), but did not differ with evidence of *Mtb* infection or with pulmonary and extrapulmonary TB (Additional file: Fig. S3A-B).

CFP-10pep signal more accurately distinguished TB and unlikely TB than *Mtb* culture (83.8% versus 61.0%, *p* < 0.001), AFB smear (82.8% versus 55.7%, *p* < 0.001), TST (91.7% versus 80.3%, *p* = 0.007), and chest X-ray (84.1% versus 79.7%, *p* = 0.015) results in infants with each result (Table 5). Serum CFP-10pep detected 45 TB cases not identified by culture or smear (Fig. 2b) and had higher sensitivity for pulmonary and extrapulmonary TB than culture or smear (Fig. 2c, d).

**Table 5:** TB diagnostic performance of CFP-10pep, smear, culture, and TST

Comparison	Participants^**a**^	CFP-10 Sensitivity	2^nd^ assay Sensitivity	***p*** value^**b**^	CFP-10 Specificity	2^nd^ assay Specificity	***p*** value	CFP-10 Accuracy	2^nd^ assay Accuracy	***p*** value
**CFP-10pep vs X-ray**	183	82.3% (81.9–82.7)	88.2% (87.9–88.5)	0.346	85.2% (85.0–85.4)	74.7% (74.4–75.1)	0.019	84.1% (84.0–84.2)	79.7% (79.6–79.9)	0.015
**CFP-10pep vs smear**	140	85.9% (85.5–86.3)	15.6% (15.2–16.0)	< 0.001	80.2% (79.8–80.6)	89.4% (89.2–89.7)	0.127	82.8% (82.6–83.0)	55.7% (55.3–56.0)	< 0.001
**CFP-10pep vs culture**	136	88.3% (87.9–88.6)	11.6% (11.3–12.0)	< 0.001	80.2% (79.8–80.6)	100% (95.3–100)	< 0.001	83.8% (83.6–84.0)	61.0% (60.6–61.37)	< 0.001
**CFP-10pep vs TST**	377	81.8% (81.3–82.2)	68.1% (67.5–68.8)	0.083	93.8% (93.8–93.9)	82.9% (82.8–83.0)	< 0.001	91.7% (91.7–91.8)	80.3% (80.2–80.4)	0.007

The results listed in this Table indicate percent diagnostic sensitivity, specificity and accuracy with an estimated 95% CI adjusted for sample size

^a^Participants who were conducted both CFP-10pep assay and secondary assay (X-ray, smear, culture, and TST, respectively)

^b^Listed *p* values indicate the probability for a significant difference between the diagnostic sensitivity, specificity, or accuracy of CFP-10 and the test listed in the first column of the matching row by McNemar’s test

### CFP-10pep for early TB detection and treatment response

**Fig. 3:**
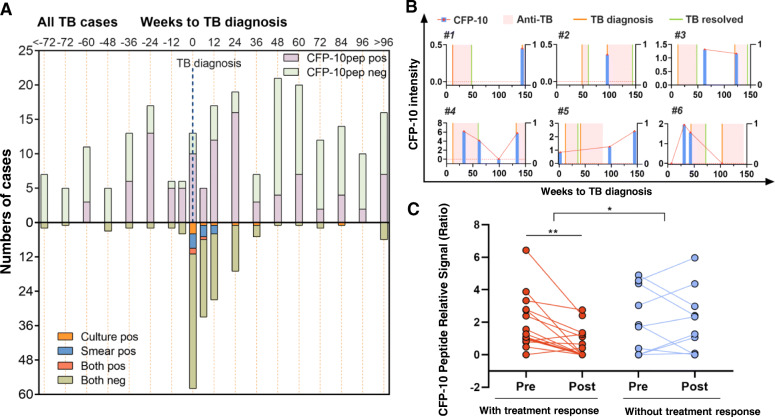
Serum CFP-10pep assay in early detection, recurrent TB, and TB treatment monitoring. **a** Distribution of CFP-10pep-positive and CFP-10pep-negative signal (above the *x*-axis) and culture and/or smear positive and culture + smear negative results (below the *x*-axis) in total TB cases, where time zero (vertical dashed line) denotes time of TB diagnosis. Only one sample per child was available and evaluated in each 12-week interval. **b** Recurrent TB cases. The orange vertical lines denote time of TB diagnosis relative to study enrollment, the pink box indicates the duration of an anti-TB treatment, and the vertical green lines indicate the time of TB resolution, while the red dots and blue bar connected by red lines indicate the time and intensity of serum CFP-10pep signal. **c** Serum CFP-10pep signal changes from pre-diagnosis (pre) to post-diagnosis (post) in TB cases with (red dots) and without (blue dots) positive anti-TB treatment responses. Connected points indicate the magnitude and time frame of changes for individual patients. **p < 0.01 by paired *t* test, *p < 0.05 by repeated measure mixed model to compare the paired difference between TB cases with and without positive anti-TB treatment responses

Most TB cases with pre-diagnosis serum had at least one CFP-10pep-positive sample prior to diagnosis (60%; 32/53), with positive samples clustering near diagnosis, but detectable by 60 weeks before diagnosis (median 18 weeks pre-diagnosis) (Fig. 3a). CFP-10pep detection rates tended to be higher in HIV-infected TB cases, but similar detection time courses were observed regardless of HIV infection status (Additional file: Fig. S3C-D). Overall, CFP-10pep positive sample frequency rose to 46% by 36 weeks pre-diagnosis, ranged between 76–83% and 75–100% in the 24-week intervals pre- and post-diagnosis, respectively and then decreased to 18–43% (Additional file: Fig. S4A). Culture and smear positive rates were low (12% and 13%) at diagnosis and zero by 48 weeks post-treatment initiation. Similar results were observed for HIV-infected and HIV-uninfected infants (Additional file: Fig. S4B-C). Six infants had recurrent TB with 5- to 96-week intervals between first TB resolution and second TB diagnosis (Fig. 3b). Five were CFP-10pep-positive at or after disease recurrence (Fig. 3b; #1–5), including one patient who completed treatment without recorded evidence for a cure or continued TB disease (Fig. 3b, #5).

Most TB cases identified and treated under the P1041 protocol were also identified by 2015 NIH criteria (91%; 106/117) and had positive treatment responses (66%; 70/106) that did not differ by HIV status (Tables 1, 62% vs. 70%, *p* > 0.05). Among those with pre- and post-diagnosis serum, 60% (15/25) responded to treatment and tended to exhibit CFP-10pep decreases not observed in non-responders (Fig. 3c). CFP-10pep signal also increased near TB diagnosis, and decreased to non-detectable levels during or after anti-TB treatment, consistent with clinical evaluation of disease resolution (Additional file: Fig. S5).

## Discussion

This study extends our previous research employing serum CFP-10pep assays to diagnose TB in adults [18, 22, 23], indicating that it can effectively diagnose TB in HIV-infected and HIV-uninfected infants, including unconfirmed TB cases missed by sputum-based methods. Our data also suggests its potential utility for diagnosis of nascent TB cases and monitoring treatment responses.

The World Health Organization (WHO) recommends that non-sputum biomarker tests have sensitivities ≥ 66% for children with intrathoracic confirmed TB cases to match Xpert MTB/RIF sensitivity [26]. CFP-10pep demonstrated 100% (95% CI, 54–100%) and 81% (95% CI, 69–90%) sensitivity for confirmed and unconfirmed TB and unlike most other TB diagnostics did not differ by HIV status, in agreement with previous results with adult TB cases [18, 22, 26]. Serum CFP-10pep signal was detected in most unconfirmed TB cases (> 80%; 51/63), and all (5/5) extrapulmonary TB cases, including TB lymphadenitis and TB meningitis cases missed by respiratory sampling.

Serum CFP-10pep exhibited 95% overall specificity (95% CI 92–97%), approaching the 98% specificity threshold recommended by the WHO, but varied among unlikely TB subgroups, ranging from 85.7 to 88.6% in non-tuberculous mycobacteria (NTM) and latent TB infection subgroups and from 97.8 to 100% in subgroups without and with other disease (Table 4). The two false positives NTM CFP-10pep results matched a *Mycobacterium kansasii* infection, which could express a CFP-10 ortholog, and a *Mycobacterium avium* and bacterial/fungal co-infection. Most CFP-10pep false positives in the latent TB subgroup (4 of 5) were detected 6 to 60 weeks before a positive TST and could indicate nascent *Mtb* infections that subsequently resolved to latent TB infections. Notably, most unlikely TB cases with CFP-10pep positive serum (73%) met at least one criterion for unconfirmed TB diagnosis, suggesting that some could have had subclinical disease. CFP-10pep signal might thus identify individuals who might benefit from anti-TB intervention, particularly in populations where latent TB cases do not routinely receive anti-TB treatment.

Delayed anti-TB treatment initiation, especially for children with HIV/TB co-infections or disseminated TB (including TB meningitis), may result in irreversible damage or death [2]. Serum CFP-10pep was detected up to 60 weeks before TB diagnosis, and identified TB with 77% sensitivity 24 weeks prior to TB-diagnosis, with detection rates increasing as sample collection times approached approach TB diagnosis. Most positive samples were obtained from HIV-infected children who were analyzed more frequently and might exhibit greater antigenemia due to reduced *Mtb* containment, but the utility of CFP-10 pep as a biomarker for initial *Mtb* infection or nascent TB deserves further study.

Current treatment guidelines indicate that drug susceptible TB cases should receive 6–9 months of anti-TB therapy, but treatment responses may vary with age, immune function, infection site, and drug resistance [27]. Overtreatment can increase adverse events, while inadequate treatment can promote TB recurrence and drug resistance. Rapid monitoring assays are needed to address these issues, but current microbiologic and immunologic assays lack required speed, sensitivity, and/or specificity, while Xpert MTB/RIF cannot distinguish live and dead mycobacteria, limiting its utility for treatment monitoring [28]. CFP-10pep detection rates and concentrations decreased after anti-TB treatment initiation in TB cases with clinical responses, suggesting CFP-10pep may serve as a biomarker of treatment response. Serum CFP-10pep evaluation would be particularly beneficial for groups, including infants, where sputum-derived results for diagnosis and treatment evaluation are rarely available.

This study has several limitations. First, it used cryopreserved serum from a study not designed to evaluate TB diagnostics. However, all suspected TB cases underwent extensive evaluation that allowed post-hoc assignment of current TB classifications, and longitudinal assessment pre- and post-TB evaluation. Serum samples were stored at − 80 °C for 9 to 13 years prior to CFP-10pep assay analysis. We have previously reported that CFP-10pep signal does not markedly decrease after 30 days storage at − 80 °C (90.7 ± 0.6% recovery) [ 23]. We do not, however, have data for the effect of long-term storage, which might allow CFP-10 degradation or modification to attenuate CFP-10pep detection, and underestimate the diagnostic sensitivity of the CFP-10pep assay.

Second, our study exclusion criteria may have introduced bias affecting our sensitivity and specificity estimates. Most differences between the excluded and analyzed groups were minor (Table S1) but analyzed children were more likely to have been breast-fed, had less severe HIV and CD4 classifications, and had a higher overall death rate without prior TB diagnosis, although the fraction of HIV-infected children was larger in the analyzed versus excluded population. HIV-uninfected children may have preferentially excluded for reduced sample availability, since they had fewer study visits than HIV-infected children after the intervention period (24 weeks vs 12 weeks) and thus fewer visits at which serum could be drawn and archived for subsequent analysis. Excluded HIV-uninfected children may also have been healthier overall and thus have had fewer serum samples collected overall. Notably, though, TB diagnosis frequency was higher in the analyzed versus the excluded cohort for both the HIV-infected and HIV-uninfected children.

Third, the analyzed cohort contained relatively few TB cases, most of which were diagnosed as unconfirmed TB (92.3%), reducing its ability to provide narrow confidence intervals for diagnostic sensitivity estimates, particularly for subgroup analyses. Several factors could explain the scarcity of confirmed TB cases. Infants and young children with TB frequently have paucibacillary TB and are difficult to diagnose by microbiologic methods. Our analysis cohort was also drawn from a study that employed active case finding, which can diagnose TB earlier than passive screening, often before such cases have positive microbiologic test results [29]. Our exclusion criteria did not markedly affect confirmed TB case frequency in our analyzed study population, however, since the frequency of such cases was similar in the overall group and our analyzed cohort (1.9% vs. 1.6%).

Forth, this study cannot compare serum CFP-10pep results to molecular methods (e.g., Xpert) that were not available during the initial study. However, Xpert MTB/RIF and Xpert MTB/RIF Ultra are not superior to *Mtb* culture for pediatric TB diagnosis [10, 30].Serum CFP-10pep also had similar sensitivity for all TB manifestations, including HIV/TB co-infection and extrapulmonary TB, in contrast to Xpert MTB/RIF which exhibits reduced sensitivity for these cases, although direct comparisons are still required to validate this difference.

Fifth, most children did not have serum available at their initial TB diagnosis or most of their P1041 study visits. We therefore estimated the diagnostic sensitivity of serum CFP-10pep by evaluating its detection rate in sera collected within ± 24 weeks of TB diagnosis (± 1 HIV-uninfected cohort post-intervention study visit) to increase the number of TB cases with available sample. However, this approach is expected to underestimate diagnostic sensitivity, by evaluating some samples collected before TB development and others collected after anti-TB treatment responses. Similarly, the specificity estimate required all samples be CFP-10-negative and likely underestimate the specificity that would be obtained using a single sample. The lack of comprehensive serum samples at all study visits also required that evaluation of CFP-10pep sensitivity, and CFP-10pep decreases following anti-TB treatment, employ aggregate data from all cases instead of sequential data from the same cases. However, we are not aware of any other study with longitudinal samples and diagnostic information in a similar at-risk TB population that would allow such analyses.

Finally, the serum CFP-10pep assay described in this study utilizes MALDI-TOF mass spectrometry for its readout, which may constrain its utility in resource limited settings. Serum CFP-10pep analysis approximates or exceeds the sensitivity and specificity requirements of the WHO target product profile for non-sputum-based TB diagnostics, but does not address end-user, cost, speed, or infrastructure requirements of this profile in its current incarnation. Current research focused on the development of less expensive and complicated portable mass spectrometers could allow assays to be performed in settings lacking extensive infrastructure or highly trained personnel. Alternately, central laboratory networks similar to those developed to allow Xpert MTB/RIF analyses in high endemic TB regions could increase access, particularly if simple on-site sample processing was employed to reduce shipping constraints.

This study evaluated diagnostic performance in a population of HIV-exposed infants born in a region with high TB incidence, who were therefore at high-risk for Mtb infection, rapid TB disease progression, and re-infection. It is not clear if this may have affected the diagnostic performance of our assay. Our data suggest that HIV-infection status did not markedly affect assay sensitivity and specificity, although the number of TB cases was too small to permit accurate evaluation of any such difference. It is also unclear if the cohort exclusion criteria, which disproportionally excluded HIV-uninfected children while increasing the relative percentage of TB cases independent of HIV status, introduced a bias for sicker children that may have increased the apparent sensitivity of our assay.

However, despite the listed concerns about factors that could increase or decrease assay sensitivity, our assay data indicate that serum CFP-10pep detection has robust diagnostic sensitivity for TB in very young children, including paucibacillary and early TB cases missed by microbiologic assays, and has robust specificity to exclude at-risk children without TB, including children with latent TB infection. Given the ability of this assay to address these unmet diagnostic needs, future studies are warranted to refine the diagnostic performance of this assay in additional cohorts that address these concerns.

## Conclusions

CFP-10 secretion is critical for *Mtb* pathogenesis [17], but its detection may be masked by its low abundance, protein interactions, or homology with related mycobacteria [18]. This study quantitates an immune-enriched CFP-10-spectic peptide by mass spectrometry after serum samples are digested to disrupt masking interactions [18, 22, 23]. Our results suggest that serum CFP-10pep signal could improve TB diagnosis in children, as it exceeds the WHO-specified sensitivity requirements for new non-sputum diagnostics, and exhibits similar performance for all TB manifestations, including culture-negative TB, HIV/TB co-infection, and extrapulmonary TB, which are normally challenging to diagnose. Our results also suggest that serum CFP-10pep analysis may have utility for diagnosis of nascent TB cases and TB treatment monitoring. Further analyses to validate these findings will require new study cohorts designed for biomarker analysis that employ consistent serial serum collection in well characterized patient populations. However, we believe that the apparent ability of CFP-10pep assay to directly predict, diagnose, and evaluate the therapeutic response of TB cases that are not detected and cannot be accurately monitored by available laboratory tests has great potential value for clinicians.

## Supplementary Information

**Additional file 1: Table S1.** Comparison of the baseline demographics and clinical characteristics of P1041 participants excluded and included for evaluation by the serum CFP-10pep assay. **Table S2.** The distribution of participants who received INH versus placebo by HIV status and TB category. **Table S3.** Summary of sera available per child and their distribution by group. **Table S4.** Sera distribution by group and time to TB diagnosis or diagnosis window. **Table S5.** Distribution of Unconfirmed TB evidence among included/excluded infants. **Table S6.** Distribution of Unconfirmed TB evidence and CFP-10pep assay positivity rates. **Table S7.** CFP-10pep sensitivity among children who received INH versus placebo by HIV and *Mtb* infection status. **Table S8.** CFP-10pep specificity among children who received INH versus placebo by HIV and *Mtb* infection status. **Table S9.** Comparision of the diagnostic subgroups and classification criteria used in the P1041 protocol and the 2015 NIH pediatric TB guidelines. **Table S10.** Cross-classification of analyzed children by P1041 and 2015 NIH criteria. **Fig. S1.** Age distribution at time of TB diagnosis. **Fig. S2.** Venn diagrams for the availability and TB diagnostic contribution of clinical data. **Fig. S3.** Serum CFP-10pep signal distribution by HIV infection status and TB diagnosis category and distribution of positive TB diagnostic results and CPF-10pep signal relative to TB diagnosis. **Fig. S4.** Positive proportions of CFP-10pep, culture and smear results relative to TB diagnosis time. **Fig. S5.** Changes in serum CFP-10pep levels from pre-diagnosis to post-treatment initiation in representative TB cases.

## Data Availability

The datasets used and analyzed during the current study are available from the corresponding author on reasonable request.

## Abbreviations

CFP-10pep: CFP-10 peptide
INH: Isoniazid
IS: Internal standard
MALDI-TOF: Matrix-assisted laser desorption ionization time-of-flight
*Mtb*: *Mycobacterium tuberculosis*
NTM: Non-tuberculous mycobacteria
TB: Tuberculosis
TST: Tuberculin skin tests
WHO: World Health Organization
