# Supplementary material for: Evaluation of a serum-based antigen test for tuberculosis in HIV-exposed infants: a diagnostic accuracy study
Source: BMC Med. 2021 May 18;19:113. doi: 10.1186/s12916-021-01983-w (PMC8130139; doi:10.1186/s12916-021-01983-w)
Supplement: Supplementary file 1 — Additional file 1: Table S1. Comparison of the baseline demographics and clinical characteristics of P1041 participants excluded and included for evaluation by the serum CFP-10pep assay. Table S2. The distribution of participants who received INH versus placebo by HIV status and TB category. Table S3. Summary of sera available per child and their distribution by group. Table S4. Sera distribution by group and time to TB diagnosis or diagnosis window. Table S5. Distribution of Unconfirmed TB evidence among included/excluded infants. Table S6. Distribution of Unconfirmed TB evidence and CFP-10pep assay positivity rates. Table S7. CFP-10pep sensitivity among children who received INH versus placebo by HIV and Mtb infection status. Table S8. CFP-10pep specificity among children who received INH versus placebo by HIV and Mtb infection status. Table S9. Comparision of the diagnostic subgroups and classification criteria used in the P1041 protocol and the 2015 NIH pediatric TB guidelines. Table S10. Cross-classification of analyzed children by P1041 and 2015 NIH criteria. Fig. S1. Age distribution at time of TB diagnosis. Fig. S2. Venn diagrams for the availability and TB diagnostic contribution of clinical data. Fig. S3. Serum CFP-10pep signal distribution by HIV infection status and TB diagnosis category and distribution of positive TB diagnostic results and CPF-10pep signal relative to TB diagnosis. Fig. S4. Positive proportions of CFP-10pep, culture and smear results relative to TB diagnosis time. Fig. S5. Changes in serum CFP-10pep levels from pre-diagnosis to post-treatment initiation in representative TB cases. [file 12916_2021_1983_MOESM1_ESM.docx]

**Additional file**

**Contents**

[1. Methods 2](#_Toc64847571)

[**1.1 Diagnosis of HIV and TB in the parent P1041 trial** 2](#_Toc64847572)

[**1.2 Serum CFP-10pep assay procedure** 2](#_Toc64847573)

[2. Tables 4](#_Toc64847574)

[**Table S1. Comparison of the baseline demographics and clinical characteristics of P1041 participants excluded and included for evaluation by the serum CFP-10pep assay.** 4](#_Toc64847575)

[**Table S2. The distribution of participants who received INH versus placebo by HIV status and TB category.** 5](#_Toc64847577)

[**Table S3. Summary of sera available per child and their distribution by group.** 6](#_Toc64847578)

[**Table S4. Sera distribution by group and time to TB diagnosis or diagnosis window.** 7](#_Toc64847579)

[**Table S5. Distribution of Unconfirmed TB evidence among included/excluded infants*.*** 8](#_Toc64847580)

[**Table S6. Distribution of Unconfirmed TB evidence and CFP-10pep assay positivity rates.** 9](#_Toc64847581)

[**Table S7. CFP-10pep sensitivity among children who received INH versus placebo by HIV and *Mtb* infection status.** 10](#_Toc64847582)

[**Table S8. CFP-10pep specificity among children who received INH versus placebo by HIV and *Mtb* infection status.** 11](#_Toc64847583)

[**Table S9. Comparision of the diagnostic subgroups and classification criteria used in the P1041 protocol and the 2015 NIH pediatric TB guidelines *.*** 12](#_Toc64847584)

[**Table S10. Cross-classification of analyzed children by P1041 and 2015 NIH criteria*.*** 13](#_Toc64847585)

[3. Figures 14](#_Toc64847586)

[**Figure S1. Age distribution at time of TB diagnosis*.*** 14](#_Toc64847587)

[**Figure S2. Venn diagrams for the availability and TB diagnostic contribution of clinical data.** 15](#_Toc64847588)

[**Figure S3. Serum CFP-10pep signal distribution by HIV infection status and TB diagnosis category and distribution of positive TB diagnostic results and CPF-10pep signal relative to TB diagnosis.** 16](#_Toc64847589)

[**Figure S4. Positive proportions of CFP-10pep, culture and smear results relative to TB diagnosis time.** 17](#_Toc64847590)

[**Figure S5. Changes in serum CFP-10pep levels from pre-diagnosis to post-treatment initiation in representative TB cases.** 28](#_Toc64847591)

[4. References 19](#_Toc64847592)

**1. Methods**

**1.1 Diagnosis of HIV and TB in the parent P1041 trial**

P1041 study was designed to evaluate whether primary INH prophylaxis improved TB disease-free survival among HIV-infected children or Mtb infection-free survival among HIV-uninfected children. Between December 2004 and June 2008, P1041 enrolled BCG-vaccinated infants 91-120 days-of-age with documented maternal HIV infection. Written informed consent was obtained from the legal guardians of the infants before infants underwent randomization. Exclusion criteria included infant contact with a microbiologically confirmed TB case, history of TB, evidence of failure to thrive, recurrent pneumonia, chronic diarrhea or any immunosuppressive condition besides HIV infection, or maternal anti-TB treatment at the time of the infant’s birth. ^1^

Eligible participants were prospectively classified for the parent clinical trial as HIV-uninfected and HIV-infected groups on the basis of their initial PCR assay results. Infants with initially negative HIV PCR results were retested by PCR at 24 weeks-of-age, and by ELISA at 72 weeks-of-age when a final assignment of HIV-uninfected was made if both of these tests were negative.^1^

During the parent P1041 study, participants were screened for TB risks and symptoms at each study visit with an algorithm based on South African guidelines at the time including weeks of illness (including cough), nutritional status, family history of TB, TST, fever not responding to treatment, and confirmed or suspected extrapulmonary TB (EPTB).^2^ Infants with a score of 4 or more or if presenting with clinical or radiographic evidence of pneumonia were assessed further with TB investigations including TST, CXR, microbiologic or histopathologic evaluation, as well as gastric washings or induced sputa with AFB smear and culture. CXR data was evaluated for overall quality, and CXR data with acceptable quality were evaluated for abnormalities consistent with TB (i.e., hilar lymphadenopathy; paratracheal lymphadenopathy, alveolar consolidation, miliary patterns, lung parenchymal breakdown/ cavitation, and Ghon foci) by three independent experts blinded to other clinical results associated with these infants. In cases of discordant classifications for the P1041 study, meetings would be convened to achieve a consensus classification. For the parent P1041 study, infants categorized as having protocol-defined TB were started on anti-TB treatment, as were children who did not meet these criteria but who were classified as having “non-algorithm tuberculosis” by clinical experts. All TB cases defined by P1041 protocol and “non-algorithm tuberculosis” were evaluated for their clinical response to anti-TB treatment every 3 months for the duration of their treatment or their enrollment in the study. In subsequent analyses children were retrospectively assigned to NIH TB categories based on a 2012 NIH-sponsored expert consensus panel which included Confirmed, Probable, Possible, and Unlikely TB categories.^3-4^ This 2012 NIH consensus was further revised in 2015, essentially collapsing Probable and Possible TB into one category of Unconfirmed TB. For the analyses performed in this report, infants were retrospectively assigned to NIH TB categories based on criteria stated in report by a 2015 NIH-sponsored expert consensus panel,^5^ with differences between the P1041 protocol and 2015 NIH consensus criteria and their effects on the classification of study participants outlined in **additional file: table S9** and **additional file: table S10**.

For the current study, participants were retrospectively assigned TB classifications based on these 2015 NIH criteria for pulmonary TB.^5^ Confirmed TB required microbiologic confirmation with culture. Unconfirmed TB was assigned in the absence of bacteriological confirmation if the child had at least 2 of the following: symptoms/signs suggestive of TB (persistent cough > 2 weeks), weight loss/failure to thrive, persistent (>1 week) or unexplained fever, persistent unexplained lethargy or reduced playfulness), chest radiograph consistent with TB, close TB exposure or immunologic evidence of M. tuberculosis infection (for this study tuberculin skin test (TST), induration of ≥ 5 mm for HIV-infected infants and ≥ 10 mm for HIV-uninfected infants), or positive clinical response to TB treatment. Unlikely TB was assigned when bacteriological confirmation was not obtained and criteria for Unconfirmed TB were not met; Children classified as Unlikely TB may have not further testing for TB or other conditions based on their study evaluations (healthy with no evidence of disease), or may have exhibited evidence of other infections or conditions (e.g., non-tuberculous mycobacteria infection; bacterial, viral or fungal infection; latent TB infection; or an inflammatory condition), as detailed in **table 4**. Both the Unconfirmed TB and Unlikely TB groups were segregated into subgroups with or without immunological evidence of *Mtb* infection.

**1.2 Serum CFP-10pep assay procedure**

Cryopreserved sera from eligible children for current study were analyzed using a previously reported assay that employs nanoparticle-based immune-enrichment and MALDI-TOF mass spectrometry steps to detect and quantify sequence-specific peptides of the *Mtb* virulence factors CFP-10 and ESAT-6 in trypsin digested aliquots of standard serum (no additives) or EDTA plasma samples.^6^ Since ESAT-6 was detected in only a fraction of TB cases and rarely detected in the absence of CFP-10 in previous studies,^7-8^ we did not analyze its diagnostic ability in the current study.

Briefly, 1mL of ~7 x10^8^ functionalized NanoDisks (1,000 × 400-nm discs with 40-nm pores coated with a thin silica layer)^6^ were vacuum-dried, suspended with 1 mL PBS buffer containing 20 μg/mL of a CFP-10-specific peptide antibody (anti-1593.75) for 2 h at 25 °C, then centrifuged for 5 min at 10,000 × g^6-8^. After aspirating the supernatant, NanoDisks were suspended in 1 mL of a solution of 200 mM Tris (pH 7) and 100 mM NaCl and incubated for 30 min at 25 °C. These antibody-conjugated NanoDisks were then washed three times with phosphate-buffered saline, suspended in 60 μL of PBS and stored at 4 °C for < 1 week until use.

Aliquots (100 μL) of participant serum were added to 400 μL of 100 mM NH_4_HCO_3_, mixed with 10 μL of 1 mg/mL sequencing-grade modified trypsin (Promega), and microwave-irradiated for 20 min at 1200 W in a 1000-1080 mL water bath, then adjusted to a final concentration of 0.1% trifluoroacetic acid to achieve a neutral PH. Digested serum samples were then supplemented with 10 nM of a stable-isotope-labeled internal standard (IS) peptide (m/z 1,603.60; GenScript USA), and mixed with 10 μL of antibody-conjugated NanoDisks for 2 h at 25°C, after which the NanoDisks were pelleted by 5 min centrifugation at 10,000 × g. NanoDisks pellets were washed three time ﻿with 1 mg/mL 1,2-dioleoyl-sn-glycero-3-phospho-L-serine (Avanti Polar Lipids), and suspended in 6 μL of deionized water, after which 4.5 μL (1.5 μL × 3) was analyzed by MALDI-TOF-MS.

The calibration curve used for CFP-10pep quantification was generated by spiking healthy commercial serum aliquots with serial dilutions of recombinant protein and a constant amount of a stable-isotope-labeled internal standard peptide (IS) with the same sequence as the target CFP-10pep peptide. All analyzed serum samples were spiked with IS to allow quantification of detected CFP-10pep signal, as CFP-10pep concentration in clinical serum samples was determined by evaluation their CFP-10pep-to-IS ratio against the calibration curve. This approach demonstrates within-run precision (%CV) varying from14.4% to 19.7%, between-run precision ranging from 16.3% to 15.2%, and accuracy varying from 91.5% (20nM) to 78.5%, as CFP10pep concentration was decreased from 20nM to 1 nM.^6^

**2. Tables**

**Table S1. Comparison of the baseline demographics and clinical characteristics of P1041 participants excluded and included for evaluation by the serum CFP-10pep assay.**

| **Number (%)** | **Not Included in CFP-10pep evaluation** | | | **Included in CFP-10pep evaluation** | | |
| --- | --- | --- | --- | --- | --- | --- |
|  | Total | HIV-infected^a^ | HIV-uninfected | Total | HIV-infected^b^ | HIV-uninfected |
|  | 832 | 269 (32.3) | 563 (67.7) | 519 | 284 (54.7) | 235 (45.3) |
| **Age – days** | | | | | | |
| **Median** | 97 | 96 | 98 | 94^ǂ^ | 95 | 93^ǂ^ |
| **Range** | 91 to 120 | 91 to 120 | 91 to 120 | 91 to 120 | 91 to 120 | 91 to 120 |
| **WHO Weight-for-age z score** | | | | | | |
| **Median** | -0.44 | -1.17 | -0.13 | -0.77 **ǂ** | -1.35 | -0.33 |
| **Range** | -6.09 to 3.26 | -6.09 to 2.63 | -3.66 to 3.26 | -5.84 to 3.48 | -5.84 to 3.48 | -4.96 to 1.98 |
| **Male sex – no. (%)** | 406 (51.2) | 124 (46.1) | 282 (50.1) | 242 (46.6) | 116 (40.8) | 126 (53.6) |
| **Race or ethnic group; # (%)** | | | | | | |
| **Indigenous African** | 794 (95.4) | 259 (96.3) | 535 (95.0) | 517 (99.6) **ǂ** | 283 (99.6) **ǂ** | 234 (99.6) **ǂ** |
| **Mixed ancestry or other** | 38 (4.6) | 10 (3.7) | 28 (5.0) | 2 (0.4) | 1 (0.4) | 1 (0.4) |
| **Breast-feeding; # (%)** | | | | | | |
| **Ever breast-fed** | 60 (7.2) | 33 (12.3) | 27 (4.8) | 61 (11.7) **ǂ** | 42 (14.8) | 19 (8.1) |
| **Breast-fed at baseline** | 11 (1.3) | 7 (2.6) | 4 (0.7) | 25 (4.8) **ǂ** | 22 (7.7) **ǂ** | 3 (1.3) |
| **Parent study arm; # (%)** | | | | | | |
| **Isoniazid** | 413 (49.6) | 125 (46.5) | 288 (51.2) | 263 (50.7) | 150 (52.8) | 113 (48.1) |
| **Placebo** | 419 (50.4) | 144 (53.5) | 275 (48.8) | 256 (49.3) | 134 (47.2) | 122 (51.9) |
| **Maternal history of TB; # (%)** | 68 (8.2) | 26 (9.7) | 42 (7.5) | 28 (5.4) | 13 (4.6) **ǂ** | 15 (6.8) |
| **During index pregnancy** | 6 (0.7) | 1 (0.4) | 5 (0.9) | 2 (0.4) | 1 (0.4) | 1(0.4) |
| **Before index pregnancy** | 62 (7.5) | 25 (9.3) | 37 (6.6) | 26 (5.0) | 12 (4.2) **ǂ** | 14 (6.4) |
| **CDC clinical HIV category; # (%)^c^** | | | | | | |
| **N (asymptomatic)** | ·· | 131 (50.8) | ·· | ·· | 223 (79.6) **ǂ** | ·· |
| **A (mildly symptomatic)** | ·· | 99 (38.4) | ·· | ·· | 43 (15.4) | ·· |
| **B (moderately symptomatic)** | ·· | 23 (8.9) | ·· | ·· | 14 (5.0) | ·· |
| **CD4+ percentage^c^** | | | | | | |
| **Median** | ·· | 27 | ·· | ·· | 30 **ǂ** | ·· |
| **Range** | ·· | 6 to 52 | ·· | ·· | 6 to 58 | ·· |
| **CD4+ percentage; # (%) ^c^** | | | | | | |
| **<20%** | ·· | 59 (23.0) | ·· | ·· | 52 (19.7) **ǂ** | ·· |
| **20%-24%** | ·· | 53 (20.7) | ·· | ·· | 33 (12.5) | ·· |
| **25-34%** | ·· | 84 (32.8) | ·· | ·· | 104 (39.4) | ·· |
| **≥35%** | ·· | 60 (23.4) | ·· | ·· | 75 (28.4) | ·· |
| **Plasma HIV-1 RNA at Entry; copies/mL** | | | | | | |
| **Median** | ·· | 750,000 | ·· | ·· | 527,000 **ǂ** | ·· |
| **Range** | ·· | ≤400->750,000 | ·· | ·· | ≤400 to >750,000 | ·· |
| **Infant Outcomes** | | | | | | |
| **Protocol-defined TB ^d^** | 36 (4.3) | 19 (7.1) | 17 (3.0) | 92 (17.7) | 50 (17.6) | 42 (17.9) |
| **Death without prior TB** | 32 (3.8) | 30 (11.2) | 2 (0.4) | 8 (1.5) | 6 (2.1) | 2 (0.9) |

^a^ Four excluded participants and ^b^ two included participants who tested HIV-negative at entry but HIV-positive after enrollment, were classified as HIV-infected.

^c^ HIV-uninfected participants who were missing data at P1041 study entry were excluded from these statistics.

^d^ P1041 protocol definitions were applied in this table excluding Non-algorithm TB and latent TB. Differences between P1041 protocol and 2015 NIH consensus criteria and their effects on the classification of study participants are indicated in additional files Table S10 and Table S11.

**ǂ** Denotes p-values <0.05 for differences between matching groups of excluded P1041 study participants and those individuals included in the current study.

The included population had proportionally more HIV-infected infants, who had relatively more asymptomatic disease, lower plasma HIV-1 RNA levels, higher CD4+ cell percentages, and reduced exposure to maternal TB. Positivity rates for all TB-related parameters were similar between HIV-infected and HIV-uninfected infants within the TB and Unlikely TB groups, with the exception of an increased TST positivity rate in HIV-infected vs. HIV-uninfected infants with Unlikely TB, and differed between infants with TB and Unlikely TB in each group, with the exception of TB contact exposure and AFB smear results.

**Table S2. The distribution of participants who received INH versus placebo by HIV status and TB category.**

|  | **Total ***  **(N=519)** | | | **HIV-infected**  **(N=284)** | | | **HIV-uninfected**  **(N=235)** | | |
| --- | --- | --- | --- | --- | --- | --- | --- | --- | --- |
|  | **INH**  **(N=263)** | **Placebo**  **(N=256)** | **P value** | **INH**  **(N=150)** | **Placebo**  **(N=134)** | **P value** | **INH**  **(N=113)** | **Placebo**  **(N=122)** | **P value** |
| **TB cases – no. (%)** | 56 | 61 | 0.49 | 33 | 33 | 0.67 | 23 | 28 | 0.64 |
| **Confirmed PTB** | 3 | 3 | 0.27 | 2 | 1 | 0.15 | 1 | 2 | 0.99 |
| **Confirmed EPTB ^a^** | 0 | 2 | ·· | 0 | 2 | ·· | 0 | 0 | ·· |
| **Unconfirmed PTB** | 53 | 55 | ·· | 31 | 29 | ·· | 22 | 26 | ·· |
| MTB infection **^b^** | 30 | 43 | 0.02 | 18 | 27 | <0.001 | 12 | 16 | 0.77 |
| No MTB infection **^c^** | 23 | 12 | ·· | 13 | 2 | ·· | 10 | 10 | ·· |
| **Unconfirmed EPTB ^d^** | 0 | 3 | ·· | 0 | 3 | ·· | 0 | 0 | ·· |
| **Unlikely TB – no. (%)** | 207 | 195 | ·· | 117 | 101 | ·· | 90 | 94 | ·· |
| MTB infection | 23 | 30 | 0.21 | 13 | 19 | 0.13 | 10 | 11 | 0.99 |
| No MTB infection **^e^** | 184 | 165 | ·· | 104 | 82 | ·· | 80 | 83 | ·· |

* Two infants diagnosed with both Unconfirmed EPTB and Unconfirmed PTB were counted once in this total.

^a^ Both of these EPTB cases were diagnosed as TB lymphadenitis.

^b^ MTB infection = TST-positive (induration of ≥ 5 mm for HIV-infected infants and ≥ 10 mm for HIV-uninfected infants).

^c^ No MTB infection = TST-negative (induration of < 5 mm for HIV-infected infants and < 10 mm for HIV-uninfected infants).

^d^ Two infants were diagnosed with both Unconfirmed TB lymphadenitis and Unconfirmed PTB, and the third was diagnosed with TB meningitis.

^e^ TST-negative or no TST result available for infants not suspected of TB who did not reach a protocol-designated visit for TST performance (parent study weeks 96, 144, 196). Total and HIV-infected Unconfirmed TB cases with prior INH Prophylaxis had relatively fewer TST positive results (Mtb infection) than did their corresponding placebo groups.

**Table S3.** **Summary of sera available per child and their distribution by group.**

|  | **HIV-infected** | | | | | | | **HIV-uninfected** | | | | | | **All**  **Infants** | | **All Samples** |
| --- | --- | --- | --- | --- | --- | --- | --- | --- | --- | --- | --- | --- | --- | --- | --- | --- |
|  | Infants | Samples | # serum samples / child | | | | | Infants | Samples | # serum samples / child | | | |  |  |  |
|  |  |  | 1 | 2 | 3 | 4 | 5 |  |  | 1 | 2 | 3 | 4 |  |  | |
| **Total TB cases** | **66*** | **139** | **27** | **18** | **10** | **9** | **2** | **51** | **84** | **26** | **18** | **6** | **1** | **117** | **223** | |
| **Confirmed PTB** | 3 | 6 | 2 | ·· | ·· | 1 | ·· | 3 | 4 | 2 | 1 | ·· | ·· | 6 | 10 | |
| **Confirmed EPTB** ^a^ | 2 | 5 | ·· | 1 | 1 | ·· | ·· | ·· | ·· | ·· | ·· | ·· | ·· | 2 | 5 | |
| **Unconfirmed PTB** | 60 | 127 | 24 | 17 | 9 | 8 | 2 | 48 | 80 | 24 | 17 | 6 | 1 | 108 | 207 | |
| MTB infection **^b^** | 45 | 96 | 19 | 11 | 7 | 6 | 2 | 28 | 43 | 15 | 11 | 2 | ·· | 73 | 139 | |
| No MTB infection **^c^** | 15 | 31 | 5 | 6 | 2 | 2 | ·· | 20 | 37 | 9 | 6 | 4 | 1 | 35 | 68 | |
| **Unconfirmed EPTB ^d^** | 3 | 4 | 2 | 1 | ·· | ·· | ·· | ·· | ·· | ·· | ·· | ·· | ·· | 3 | 4 | |
| **Unlikely TB participants** | **218** | **235** | **202** | **15** | **1** | **··** | **··** | **184** | **202** | **168** | **14** | **2** | **··** | **402** | **437** | |
| MTB infection | 32 | 34 | 30 | 2 | ·· | ·· | ·· | 21 | 24 | 19 | 1 | 1 | ·· | 53 | 58 | |
| No MTB infection **^e^** | 186 | 201 | 172 | 13 | 1 | ·· | ·· | 163 | 178 | 149 | 13 | 1 | ·· | 349 | 379 | |
| **Overall participants** | **284** | **374** | **229** | **33** | **11** | **9** | **2** | **235** | **286** | **194** | **32** | **8** | **1** | **519** | **660** | |

* Two infants diagnosed with both Unconfirmed EPTB and Unconfirmed PTB were counted once in this total.

^a^ Both of these EPTB cases were diagnosed as TB lymphadenitis.

^b^ MTB infection = TST-positive (induration of ≥ 5 mm for HIV-infected infants and ≥ 10 mm for HIV-uninfected infants).

^c^ No MTB infection = TST-negative (induration of < 5 mm for HIV-infected infants and < 10 mm for HIV-uninfected infants).

^d^ Two infants were diagnosed with both Unconfirmed TB lymphadenitis and Unconfirmed PTB, and the third was diagnosed with TB meningitis.

^e^ TST-negative or no result available for infants not suspected of TB who did not reach a protocol-designated visit for TST testing (weeks 96, 144, 196).

**Table S4. Sera distribution by group and time to TB diagnosis or diagnosis window.**

|  | **HIV-infected** | | | | | | | | | | | | **HIV-uninfected** | | | | | | | | | | | | |  |  |
| --- | --- | --- | --- | --- | --- | --- | --- | --- | --- | --- | --- | --- | --- | --- | --- | --- | --- | --- | --- | --- | --- | --- | --- | --- | --- | --- | --- |
|  | Total | | Pre-window | | -24 weeks | | TB  diagnosis | | +24 weeks | | Post-window | | Total | | Pre-window | | -24 weeks | | TB  diagnosis | | + 24 weeks | | | Post-window | | **All Infants** | **All**  **Samples** |
|  | # infants : # samples | | | | | | | | | | | | # infants : # samples | | | | | | | | | | | | |  |  |
| **Total TB cases** | **66*** | **139** | **21** | **28** | **21** | **22** | **9** | **9** | **28** | **28** | **40** | **52** | **51** | **84** | **13** | **13** | **7** | **7** | **4** | **4** | **13** | **13** | **39** | | **47** | **117** | **223** |
| **Confirmed PTB** | 3 | 6 | ·· | ·· | 2 | 2 | ·· | ·· | 1 | 1 | 1 | 3 | 3 | 4 | ·· | ·· | ·· | ·· | ·· | ·· | 1 | 1 | 2 | | 3 | 6 | 10 |
| **Confirmed EPTB ^a^** | 2 | 5 | ·· | ·· | 1 | 1 | 1 | 1 | 2 | 2 | 1 | 1 | ·· | ·· | ·· | ·· | ·· | ·· | ·· | ·· | ·· | ·· | ·· | | ·· | 2 | 5 |
| **Unconfirmed PTB** | 60 | 127 | 21 | 28 | 19 | 19 | 8 | 8 | 24 | 24 | 38 | 48 | 48 | 80 | 13 | 13 | 7 | 7 | 4 | 4 | 12 | 12 | 37 | | 44 | 108 | 207 |
| MTB infection ^b^ | 45 | 96 | 17 | 22 | 11 | 11 | 7 | 7 | 18 | 18 | 29 | 38 | 28 | 43 | 7 | 7 | 4 | 4 | 3 | 3 | 7 | 7 | 19 | | 22 | 73 | 139 |
| No MTB infection ^c^ | 15 | 31 | 4 | 6 | 7 | 8 | 1 | 1 | 6 | 6 | 9 | 10 | 20 | 37 | 6 | 6 | 3 | 3 | 1 | 1 | 5 | 5 | 18 | | 22 | 35 | 68 |
| **Unconfirmed EPTB ^d^** | 3 | 4 | ·· | ·· | ·· | ·· | ·· | ·· | 2 | 2 | 1 | 2 | ·· | ·· | ·· | ·· | ·· | ·· | ·· | ·· | ·· | ·· | ·· | | ·· | 3 | 4 |
| **Unlikely TB participants** | **218** | **235** | **133** | **143** | **92** | **92** | **··** | **··** | **··** | **··** | **··** | **··** | **184** | **202** | **138** | **154** | **48** | **48** | **··** | **··** | **··** | **··** | **··** | | **··** | **402** | **437** |
| MTB infection | 32 | 34 | 24 | 25 | 9 | 9 | ·· | ·· | ·· | ·· | ·· | ·· | 21 | 24 | 16 | 18 | 6 | 6 | ·· | ·· | ·· | ·· | ·· | | ·· | 53 | 58 |
| No MTB infection^e^ | 186 | 201 | 109 | 118 | 83 | 83 | ·· | ·· | ·· | ·· | ·· | ·· | 163 | 178 | 122 | 136 | 42 | 42 | ·· | ·· | ·· | ·· | ·· | | ·· | 349 | 379 |
| **Overall participants** | **284** | **374** | **154** | **171** | **113** | **114** | **9** | **9** | **28** | **28** | **40** | **52** | **235** | **286** | **151** | **167** | **55** | **55** | **4** | **4** | **13** | **13** | **39** | | **47** | **519** | **660** |

* Two infants diagnosed with both Unconfirmed EPTB and Unconfirmed PTB were counted once in this total.

^a^ Both of these EPTB cases were diagnosed as TB lymphadenitis.

^b^ MTB infection = TST-positive (induration of ≥ 5 mm for HIV-infected infants and ≥ 10 mm for HIV-uninfected infants).

^c^ No MTB infection = TST-negative (induration of < 5 mm for HIV-infected infants and < 10 mm for HIV-uninfected infants).

^d^ Two infants were diagnosed with both Unconfirmed TB lymphadenitis and Unconfirmed PTB, and the third was diagnosed with TB meningitis.

^e^ TST-negative or no result available for infants not suspected of TB who did not reach a protocol-designated visit for TST testing (weeks 96, 144, 196).

**Table S5. Distribution of Unconfirmed TB evidence among included/excluded infants*.***

| **TB Evidence Type** | | | | **All TB cases: N (%)** | | **HIV+ TB cases: N (%)** | | **HIV- TB cases: N (%)** | |
| --- | --- | --- | --- | --- | --- | --- | --- | --- | --- |
| **TST+/TB exposed** | **CXR+** | **TB Rx response** | **TB Signs/symptoms** | Included | Excluded | Included | Excluded | Included | Excluded |
| **🗸** | **🗸** | **🗸** | **🗸** | 3 (4.8) | 3 (6.5) | 2 (4.7) | 2 (11.1) | 1 (5.0) | 1 (3.6) |
| **🗸** | **🗸** | **🗸** |  | 24 (38.1) | 19 (41.3) | 16 (37.2) | 7 (38.9) | 8 (40.0) | 12 (42.9) |
| **🗸** | **🗸** |  |  | 21 (33.3) | 18 (39.1) | 15 (34.9) | 7 (38.9) | 6 (30.0) | 11 (39.3) |
| **🗸** | **🗸** | **🗸** |  | 4 (6.3) | ·· | 3 (7.0) | ·· | 1 (5.0) | ·· |
|  | **🗸** | **🗸** | **🗸** | 6 (9.5) | 6 (13.0) | 3 (7.0) | 2 (11.1) | 3 (15.0) | 4 (14.3) |
|  | **🗸** | **🗸** | **🗸** | 3 (4.8) | ·· | 2 (4.7) | ·· | 1 (5.0) | ·· |
|  | **🗸** | **🗸** | **🗸** | 1 (1.6) | ·· | 1 (2.3) | ·· | ·· | ·· |
|  | **🗸** |  |  | 1 (1.6)* | ·· | 1 (2.3)* | ·· | ·· | ·· |
| **Total** | | | | **63** | **46** | **43** | **18** | **20** | **28** |
| **P value ^a^** | | | | p= 0.57 | | p= 0.59 | | p= 0.82 | |

* EPTB, TB meningitis with histopathology evidence.

^a^ Fisher’s exact tests comparing groups by inclusion.

**Table S6. Distribution of Unconfirmed TB evidence and CFP-10pep assay positivity rates.**

| **TB Evidence Type** | | | | **% Total cases** | | | **CFP-10+ / Total** | | | **% CFP-10 positive** | | | |
| --- | --- | --- | --- | --- | --- | --- | --- | --- | --- | --- | --- | --- | --- |
| **TST+/TB exposed** | **CXR+** | **TB Rx response** | **TB Signs/symptoms** | **All** | **HIV+** | **HIV-** | **All** | **HIV+** | **HIV-** | **All** | **HIV+** | **HIV-** |  |
| **🗸** | **🗸** | **🗸** | **🗸** | 4.8 | 4.7 | 5.0 | 3/3 | 2/2 | 1/1 | 100 | 100 | 100 |  |
| **🗸** | **🗸** | **🗸** |  | 38.1 | 37.2 | 15.0 | 17/24 | 14/16 | 3/8 | 70.8 | 87.5 | 37.5 |  |
| **🗸** | **🗸** |  |  | 33.3 | 34.9 | 30.0 | 17/21 | 11/15 | 6/6 | 81.0 | 73.3 | 100 |  |
| **🗸** | **🗸** | **🗸** |  | 6.3 | 7.0 | 5.0 | 4/4 | 3/3 | 1/1 | 100 | 100 | 100 |  |
|  | **🗸** | **🗸** | **🗸** | 9.5 | 7.0 | 15.0 | 6/6 | 3/3 | 3/3 | 100 | 100 | 100 |  |
|  | **🗸** | **🗸** | **🗸** | 4.8 | 4.7 | 5.0 | 2/3 | 1/2 | 1/1 | 66.7 | 50.0 | 100 |  |
|  | **🗸** | **🗸** | **🗸** | 1.6 | 2.3 | ·· | 1/1 | 1/1 | ·· | 100 | 100 | ·· |  |
|  | **🗸** |  |  | 1.6 | 2.3 | ·· | 1/1* | 1/1* | ·· | 100 | 100 | ·· |  |
| **Total** | | | | **100** | **100** | **100** | **51/63** | **36/43** | **15/20** | **81.0** | **83.7** | **75.0** |  |

* EPTB, TB meningitis with histopathology evidence.

**Table S7. CFP-10pep sensitivity among children who received INH versus placebo by HIV and *Mtb* infection status.**

|  | **Confirmed TB** | | **Unconfirmed TB** | | | | | |
| --- | --- | --- | --- | --- | --- | --- | --- | --- |
|  | INH | Placebo | Total | | MTB infection (TST+) | | No MTB infection (TST-) | |
|  |  |  | INH | Placebo | INH | Placebo | INH | Placebo |
| **All participants (N=63)** | | | | | | | |  |
| CFP-10pep+/total | 2/2 | 4/4 | 25/30 | 26/33 | 13/15 | 21/28 | 12/15 | 5/5 |
| Sensitivity  % (95% CI) | 100 (15.8-100) | 100 (39.8-100) | 83.3  (65.3-94.4) | 78.8  (61.1-91.0) | 86.7  (59.5-98.3) | 75  (55.1-89.3) | 80 (51.9-95.7) | 100  (47.8-100) |
| **HIV-infected participants (N=43)** | | | | | | | |  |
| CFP-10pep  +/total | 2/2 | 3/3 | 18/21 | 18/22 | 9/11 | 15/19 | 9/10 | 3/3 |
| Sensitivity  % (95% CI) | 100  (15.8-100) | 100  (29.2-100) | 85.7  (63.7-97.0) | 81.8  (59.7-94.8) | 81.8  (48.2-97.7) | 79.0  (54.4-94.0) | 90  (55.5-99.8) | 100  (29.2-100) |
| **HIV-uninfected participants (N=20)** | | | | | | | |  |
| CFP-10pep  +/total | .. | 1/1 | 7/9 | 8/11 | 4/4 | 6/9 | 3/5 | 2/2 |
| Sensitivity  % (95% CI) | .. | 100  (2.5-100) | 77.8  (40.0-97.2) | 72.7  (39.0-94.0) | 100  (39.8-100) | 66.7  (29.9-92.5) | 60  (14.7-94.7) | 100  (15.8-100) |

**Table S8. CFP-10pep specificity among children who received INH versus placebo by HIV and *Mtb* infection status.**

|  | **Unlikely TB** | | | | | |
| --- | --- | --- | --- | --- | --- | --- |
|  | Total | | MTB infection (TST+) | | No MTB infection (TST-) | |
|  | INH | Placebo | INH | Placebo | INH | Placebo |
| **All participants (N=402)** | | | | | | |
| CFP-10pep-/total | 198/207 | 182/195 | 21/23 | 26/30 | 177/184 | 156/165 |
| Specificity – % (95% CI) | 95.7  (91.9-98.0) | 93.3  (88.9-96.4) | 91.3  (72.0-98.9) | 86.7  (69.3-96.2) | 96.2  (92.3-98.5) | 94.6  (89.9-97.5) |
| **HIV-infected participants (N=218)** | | | | | | |
| CFP-10pep-/total | 111/117 | 92/101 | 12/13 | 17/19 | 99/104 | 75/82 |
| Specificity – % (95% CI) | 94.9  (89.2-98.1) | 91.1  (83.8-95.8) | 92.3  (64.0-99.8) | 89.5  (66.9-98.7) | 95.2  (89.1-98.4) | 91.5  (83.3-96.5) |
| **HIV-uninfected participants (N=184)** | | | | | | |
| CFP-10pep-/total | 87/90 | 90/94 | 9/10 | 9/11 | 78/80 | 81/83 |
| Specificity – % (95% CI) | 96.7  (90.6-99.3) | 95.7  (89.5-98.8) | 90  (55.5-99.8) | 81.8  (48.2-97.7) | 97.5  (91.3-99.7) | 97.6  (91.6-99.7) |

**Table S9. Comparision of the diagnostic subgroups and classification criteria used in the P1041 protocol and the 2015 NIH pediatric TB guidelines *.***

| **P1041 protocol-defined classification** | **2015 NIH definitions and classifications** |
| --- | --- |
| **Definite TB**:  Culture positive result | **Confirmed TB**:  Culture or Xpert positive result |
| **Probable TB**:  Positive auramine staining result plus:  1) Two or more defined TB signs or symptoms (S/S)  2) A chest x-ray (CXR) result consistent with TB | **Unconfirmed TB**:  Have at least 2 of the following:  1) S/S suggestive of TB;  2) CXR consistent with TB;  3) Close TB exposure or a positive TST/IGRA result;  4) Positive clinical response to anti-TB therapy.   - with MTB infection: positive TST/IGRA - without MTB infection: no immunological evidence |
| **Possible TB**:  1) Positive TST and CXR result consistent with TB; or  2) algorithm score≥6 by S/S, illness length, nutrition, non-responsive fever, TB exposure, suspected EPTB values |  |
| **Non-algorithm TB**:  Failed to meet protocol defined criteria but received anti-TB therapy based on the decision of the attending physicians | **Unlikely TB**:  Criteria for Confirmed and Unconfirmed TB definitions not met. Consolidated from “TB unlikely” and “Not TB with alternative diagnosis” in 2012 version NIH criteria.   - with MTB infection: positive TST/IGRA - without MTB infection: no immunological evidence |
| **Not TB**: Received an alternative diagnosis  **Latent TB**: TST positive without matching any of the above definitions |  |

**Table S10. Cross-classification of analyzed children by P1041 and 2015 NIH criteria*.***

| **P 1041 Classification** | | **2015 NIH Classification (first diagnosis)** | | | **Total** |
| --- | --- | --- | --- | --- | --- |
|  |  | TB disease | | Unlikely TB |  |
|  |  | Confirmed TB | Unconfirmed TB |  |  |
| TB disease | Definite TB | 8 | 0 | 0 | **8** |
|  | Probable TB | 0 | 13 | 2^a^ | **15** |
|  | Possible TB | 0 | 66 | 3^a^ | **69** |
| Non-algorithm TB | | 0 | 24^b^ | 7 | **31** |
| Not TB or latent TB infection | | 0 | 6 | 390 | **396** |
| **Total** | | **8** | **109** | **402** | **519** |

^a^ Because results supporting an Unconfirmed TB diagnosis by 2015 NIH criteria were beyond the diagnostic window we employed, 2 Probable and 3 Possible TB cases defined by the P1041 protocol were classified as Unlikely TB.

^b^ The P1041 protocol didn’t evaluate anti-TB treatment response. Some non-algorithm TB cases had positive clinical responses during 192-week follow-up and were classified as unconfirmed TB cases based on the 2015 NIH criteria.

**3. Figures**

**Figure S1. Age distribution at time of TB diagnosis*.***


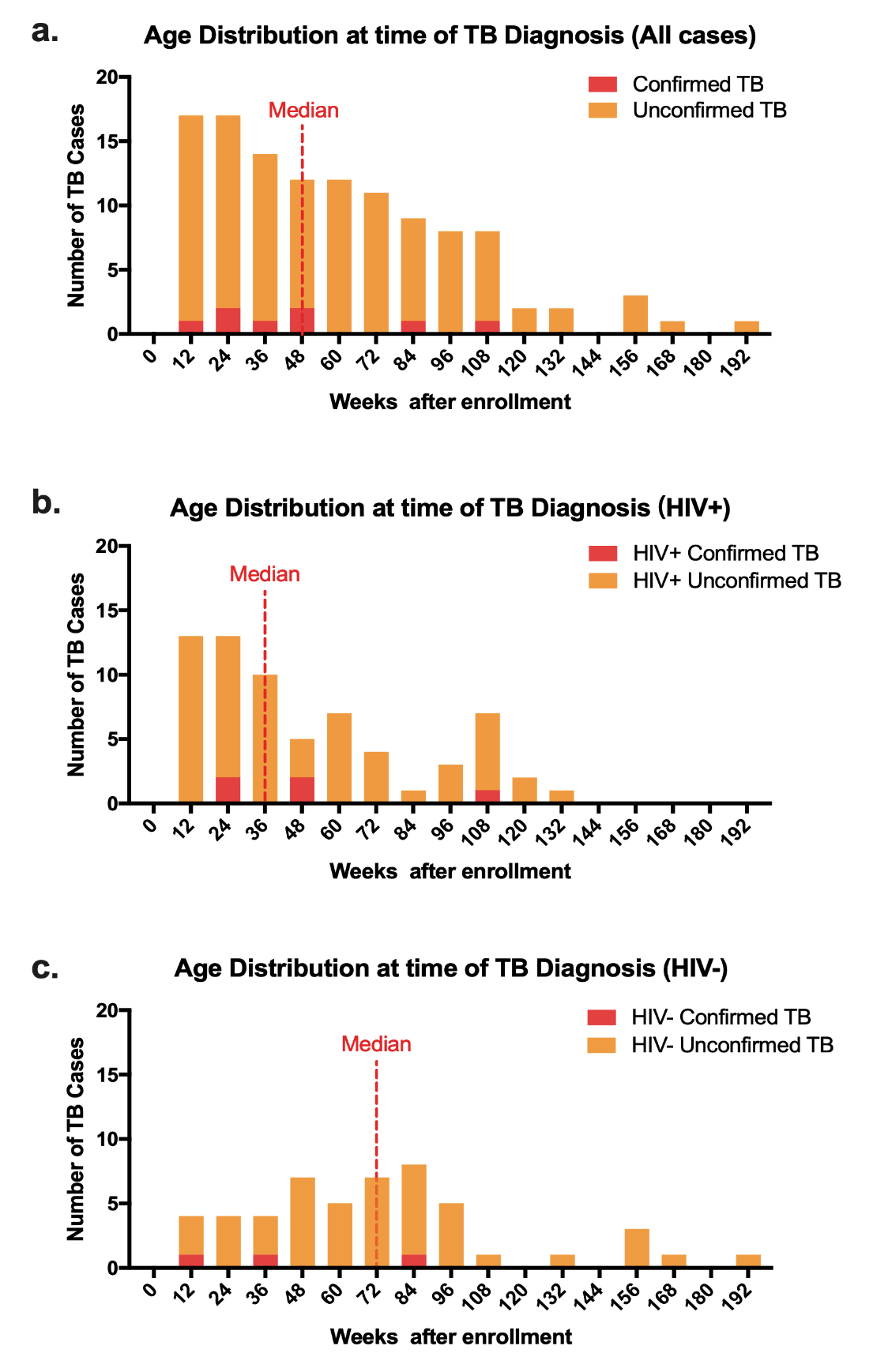
Participants were enrolled at 3-4 months age and followed for up to 192 weeks to detect TB. **A-C**) Age distribution of participants at the time of TB diagnosis among **A**) all infants, and **B**) HIV-infected and **C**) HIV-uninfected infants.

**Figure S2.** **Venn diagrams for the availability and TB diagnostic contribution of clinical data.**

Venn diagrams indicate the number of infants with TB who had the indicated combinations of test data **A)** at any point during their evaluation period (positive or negative result) or **B)** as positive contributions to their TB diagnosis. Results from two infants with TB were not depicted in this graph since these infants were not diagnosed by these tests. One exhibited positive TB signs/symptoms and anti-TB treatment response and the other revealed a positive treatment response and histopathologic evidence of TB. Infants with TB who had the indicated combinations of test data **C)** within the ±24-week window around their initial TB diagnosis and **D)** as positive contributions to their TB diagnosis within this window. Two TB cases were not included in this diagram for same reasons as stated above. Note: Some TB cases were diagnosed as unconfirmed TB first and then showed positive culture results later, leading to a higher number of infants with positive culture results than the number of infants with confirmed TB.


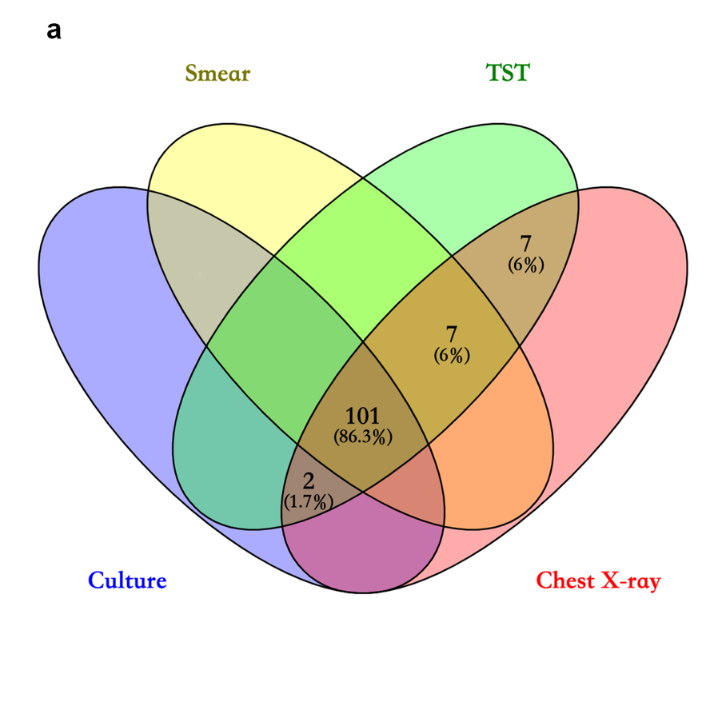

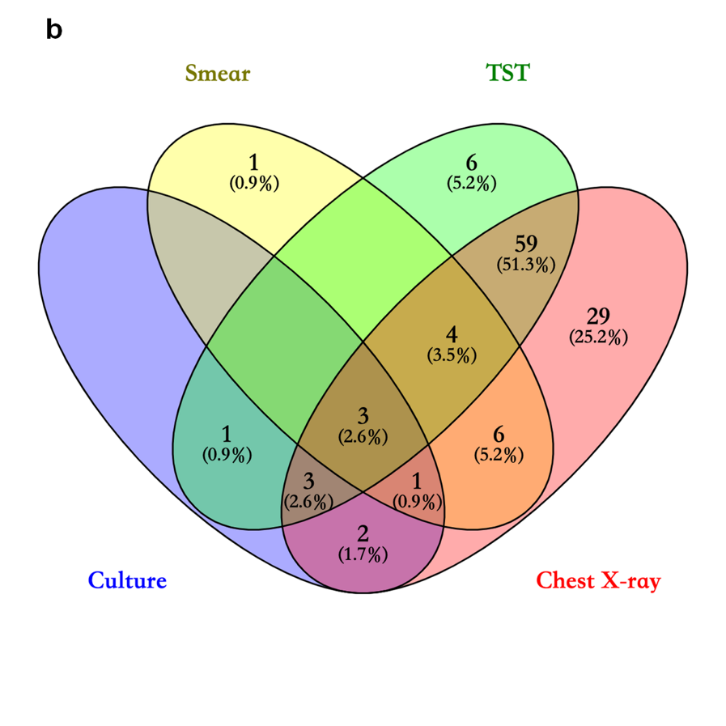

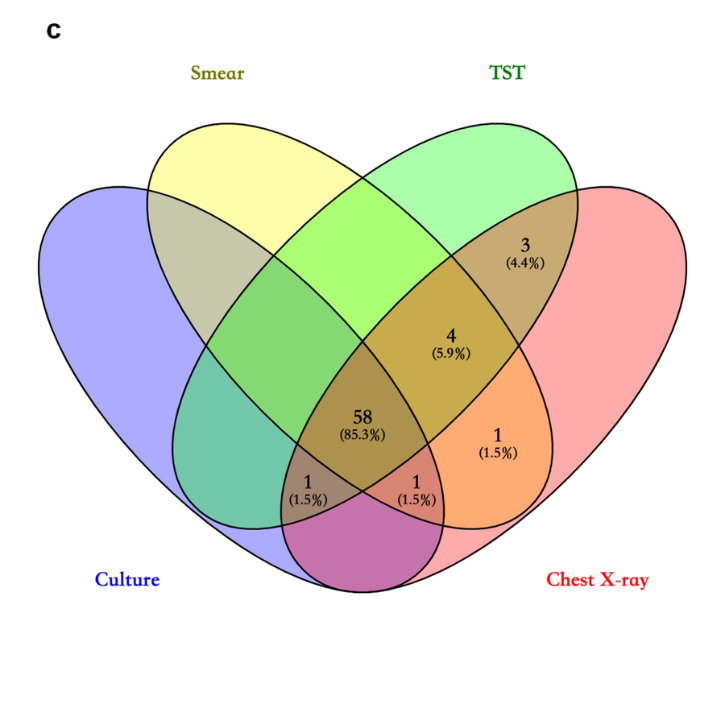

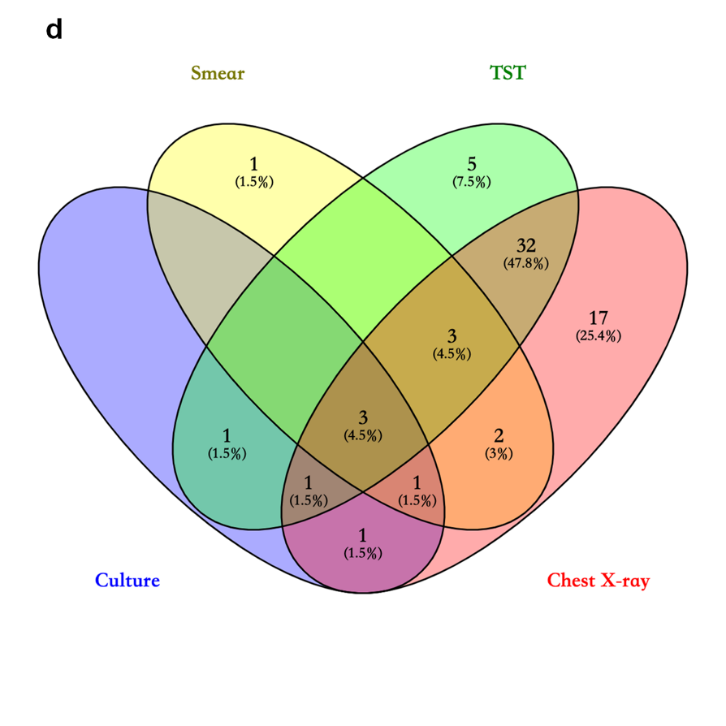


**A**

**B**

**D**

**C**

**Smear**

**TST**

**Chest X-ray**

**Culture**

**Smear**

**TST**

**Chest X-ray**

**Culture**

**Smear**

**TST**

**Chest X-ray**

**Culture**

**Smear**

**TST**

**Chest X-ray**

**Culture**

**Figure S3.** **Serum CFP-10pep signal distribution by HIV infection status and TB diagnosis category and distribution of positive TB diagnostic results and CPF-10pep signal relative to TB diagnosis.**

**A-B**) Distribution of CFP-10pep signal in **A**) HIV-infected and HIV-uninfected TB cases (Confirmed and Unconfirmed) and unlikely (UL) TG cases. **B**) CFP-10pep signal in HIV-infected and HIV-uninfected extrapulmonary TB (EPTB) and pulmonary TB (PTB) cases. Solid lines indicate group mean values and the dashed line indicates the cut-off for positive samples. **, p<0.01 by Original one-way ANOVA. **C-D**) Distribution of CFP-10-positive and CFP-10-negative signal (above the x-axis) and culture and/or smear positive and culture + smear negative results (below the x-axis) in **C**) HIV-infected and **D**) HIV-uninfected TB cases, where time zero (vertical dashed line) denotes time of TB diagnosis. Only one sample per child was evaluated in each 12-week interval when multiple samples were available.

**
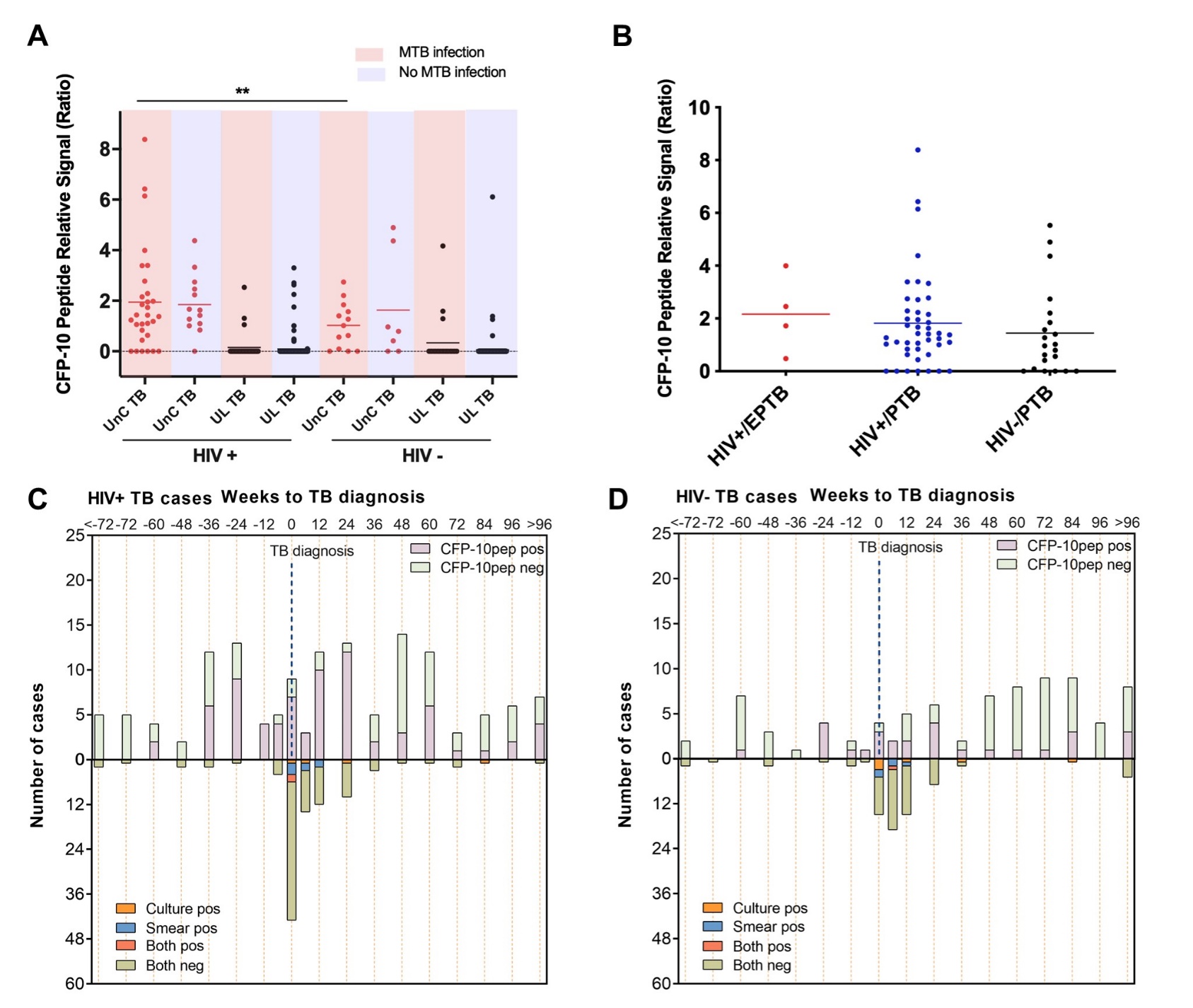
**

**Figure S4. Positive proportions of CFP-10pep, culture and smear results relative to TB diagnosis time.**

**
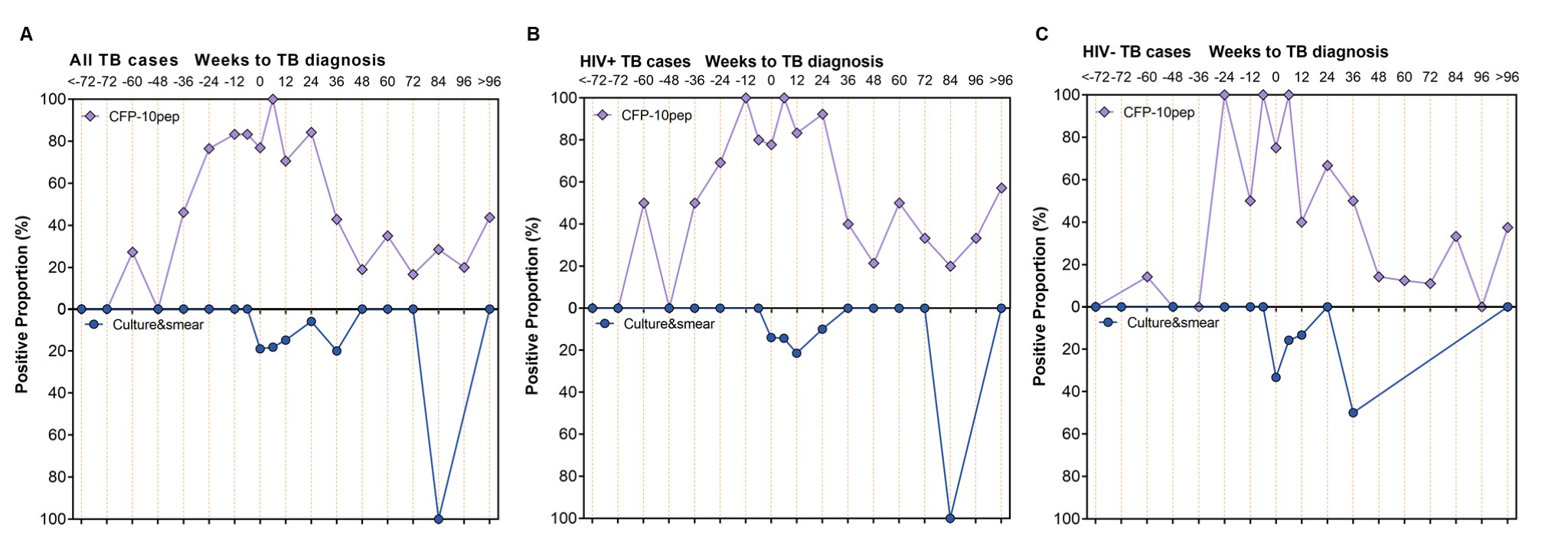
**Positive proportions of CFP-10, culture and smear in each 12-week interval among **A**) all TB cases (Confirmed and Unconfirmed) and **B**) HIV-infected and **C**) HIV-uninfected TB cases, where time zero denotes time of TB diagnosis Purple and blue lines denote the proportion of CFP-10pep-positive results and proportion of positive smear or culture results in each 12-week interval.

**Figure S5. Changes in serum CFP-10pep levels from pre-diagnosis to post-treatment initiation in representative TB cases.**

**
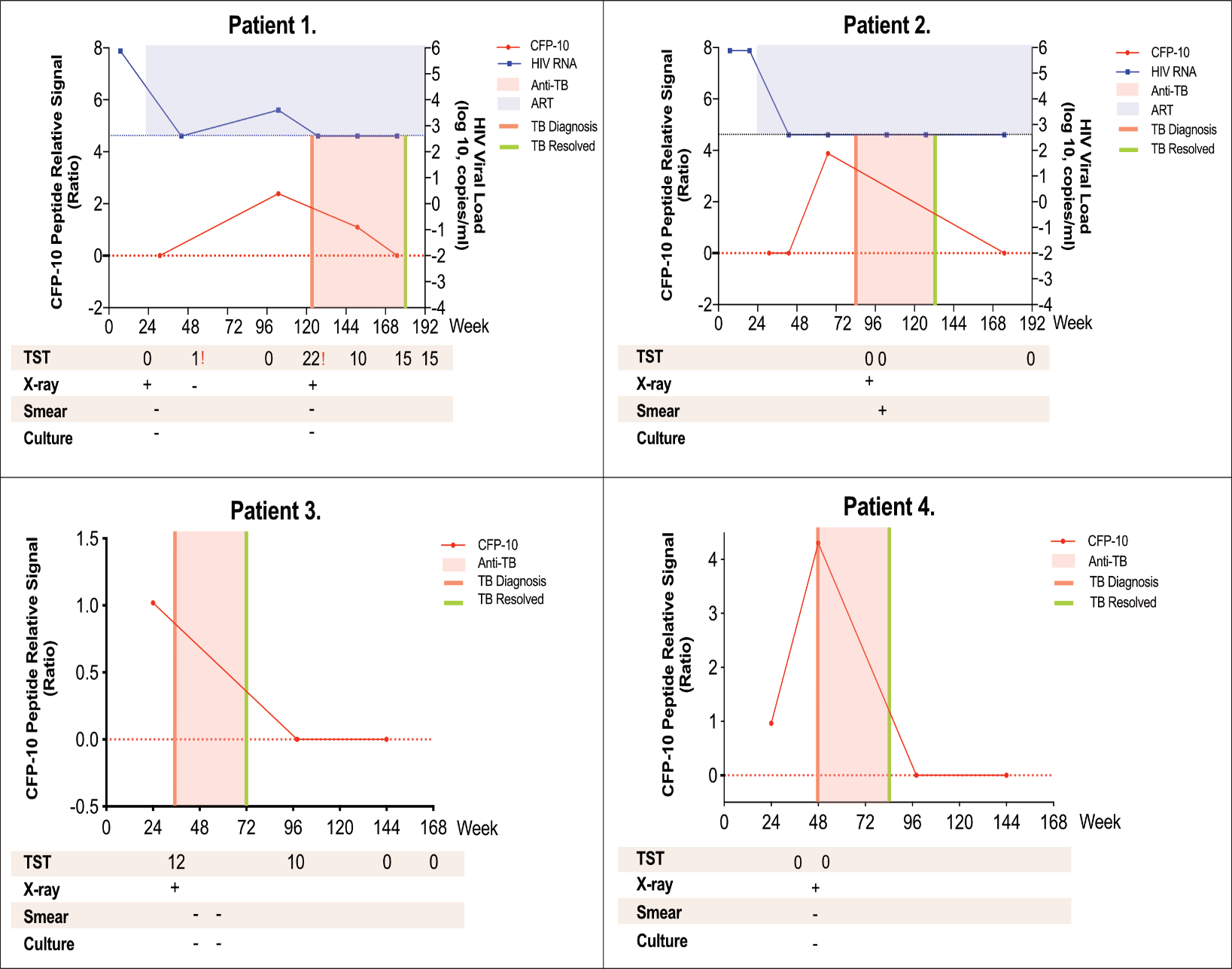
**HIV viral load (blue points) and internal-standard-normalized CFP-10pep level (red) are graphed relative to time after study enrollment in 24-week intervals in children with Confirmed TB with (top panels) or without (lower panels) HIV co-infection. Shaded boxes indicate ART (blue) and anti-TB (pink) treatment intervals, with orange line indicating initial TB diagnosis and green line indicating TB resolution. Negative (-) and positive (+) results for chest x-ray and *Mtb* smear and culture are aligned with the time of their performance below each graph. TST results indicate the diameter of the induration at the injection site in millimeters. ! Indicates known TB exposure at that time point. Results shown were chosen as representative cases from HIV-infected and HIV-uninfected children with positive responses to treatment who had the most CFP-10pep assay results and clinical data spanning the interval from pre-diagnosis to during anti-TB treatment or after its completion.

# 4. Reference

1. Madhi SA NS, Violari A, Soyeon Kim, Mark F. Cotton, Raziya Bobat, Patrick Jean-Philippe, George McSherry, and Charles Mitchell for the P1041 Study Team. Primary Isoniazid Prophylaxis against Tuberculosis in HIV-Exposed Children. N Engl J Med 2011; 365: 21-31.
2. The South African National Tuberculosis Control Programme. Practical guidelines [Internet]. 2004. http://www.kznhealth.gov.za/chrp/documents/Guidelines/GuidelinesNational/Tuberculosis/SA TB Guidelines 2004.pdf. Assessed Jan 15, 2020.
3. Beneri CA, Aaron L, Kim S, Jean-Philippe P, Madhi S, Violari A, Cotton MF, Mitchell C, Nachman S, team P. Understanding NIH clinical case definitions for pediatric intrathoracic TB by applying them to a clinical trial. Int J Tuberc Lung Dis 2016; 20: 93-100.
4. Graham SM, Ahmed T, Amanullah F, Browning R, Cardenas V, Casenghi M, Cuevas LE, Gale M, Gie RP, Grzemska M, Handelsman E, Hatherill M, Hesseling AC, Jean-Philippe P, Kampmann B, Kabra SK, Lienhardt C, Lighter-Fisher J, Madhi S, Makhene M, Marais BJ, McNeeley DF, Menzies H, Mitchell C, Modi S, Mofenson L, Musoke P, Nachman S, Powell C, Rigaud M, Rouzier V, Starke JR, Swaminathan S, Wingfield C. Evaluation of tuberculosis diagnostics in children: 1. Proposed clinical case definitions for classification of intrathoracic tuberculosis disease. Consensus from an expert panel. J Infect Dis 2012; 205: S199-208.
5. Graham SM, Cuevas LE, Jean-Philippe P, Browning R, Casenghi M, Detjen AK, Gnanashanmugam D, Hesseling AC, Kampmann B, Mandalakas A, Marais BJ, Schito M, Spiegel HM, Starke JR, Worrell C, Zar HJ. Clinical Case Definitions for Classification of Intrathoracic Tuberculosis in Children: An Update. Clin Infect Dis 2015; 61: S179-187.
6. Liu C, Zhao Z, Fan J, Lyon CJ, Wu H-J, Nedelkov D, Zelazny AM, Olivier KN, Cazares LH, Holland SM, Graviss EA, Hu Y. Quantification of circulating Mycobacterium tuberculosis antigen peptides allows rapid diagnosis of active disease and treatment monitoring. Proceedings of the National Academy of Sciences 2017; 114: 3969-3974.
7. Fan J, Zhang H, Nguyen DT, Lyon CJ, Mitchell CD, Zhao Z, Graviss EA, Hu Y. Rapid diagnosis of new and relapse tuberculosis by quantification of a circulating antigen in HIV-infected adults in the Greater Houston metropolitan area. BMC Medicine 2017; 15: 188.
8. Liu C, Lyon CJ, Bu Y, Deng Z, Walters E, Li Y, Zhang L, Hesseling AC, Graviss EA, Hu Y. Clinical Evaluation of a Blood Assay to Diagnose Paucibacillary Tuberculosis via Bacterial Antigens. Clinical Chemistry 2018; 64: 791-800.
